# Cross-linked cationic diblock copolymer worms are superflocculants for micrometer-sized silica particles†
†Electronic supplementary information (ESI) available: Macro-CTAs and the worm cross-linking chemistry; suggested mechanism for the break-up of linear worms; UV-visible spectroscopy data and assigned ^1^H NMR spectrum for the PEO_113_ macro-CTA; THF and aqueous GPC data for PEO_113_ and PQDMA_120_ macro-CTAs; kinetic data for the aqueous solution polymerization of QDMA monomer; assigned ^1^H NMR for PQDMA_125_ macro-CTA; additional TEM images and further laser diffraction traces; DLS particle size distributions; tabulated data for linear and cross-linked cationic worms diluted at pH 9 using either water or methanol; full experimental section. See DOI: 10.1039/c6sc03732a



**DOI:** 10.1039/c6sc03732a

**Published:** 2016-09-13

**Authors:** Nicholas J. W. Penfold, Yin Ning, Pierre Verstraete, Johan Smets, Steven P. Armes

**Affiliations:** a Department of Chemistry , University of Sheffield , Brook Hill , Sheffield , South Yorkshire S3 7HF , UK . Email: njwpenfopld1@sheffield.ac.uk ; Email: s.p.armes@sheffield.ac.uk; b Procter & Gamble, Eurocor NV/SA , Temselaan 100 , 1853 Strombeek-Bever , Belgium

## Abstract

A series of linear cationic diblock copolymer nanoparticles are prepared by polymerization-induced self-assembly (PISA) *via* reversible addition–fragmentation chain transfer (RAFT) aqueous dispersion polymerization of 2-hydroxypropyl methacrylate (HPMA) using a binary mixture of non-ionic and cationic macromolecular RAFT agents, namely poly(ethylene oxide) (PEO_113_, *M*_n_ = 4400 g mol^–1^; *M*_w_/*M*_n_ = 1.08) and poly([2-(methacryloyloxy)ethyl]trimethylammonium chloride) (PQDMA_125_, *M*_n_ = 31 800 g mol^–1^, *M*_w_/*M*_n_ = 1.19). A detailed phase diagram was constructed to determine the maximum amount of PQDMA_125_ stabilizer block that could be incorporated while still allowing access to a pure worm copolymer morphology. Aqueous electrophoresis studies indicated that zeta potentials of +35 mV could be achieved for such cationic worms over a wide pH range. Core cross-linked worms were prepared *via* statistical copolymerization of glycidyl methacrylate (GlyMA) with HPMA using a slightly modified PISA formulation, followed by reacting the epoxy groups of the GlyMA residues located within the worm cores with 3-aminopropyl triethoxysilane (APTES), and concomitant hydrolysis/condensation of the pendent silanol groups with the secondary alcohol on the HPMA residues. TEM and DLS studies confirmed that such core cross-linked cationic worms remained colloidally stable when challenged with either excess methanol or a cationic surfactant. These cross-linked cationic worms are shown to be much more effective bridging flocculants for 1.0 μm silica particles at pH 9 than the corresponding linear cationic worms (and also various commercial high molecular weight water-soluble polymers.). Laser diffraction studies indicated silica aggregates of around 25–28 μm diameter when using the former worms but only 3–5 μm diameter when employing the latter worms. Moreover, SEM studies confirmed that the cross-linked worms remained intact after their adsorption onto the silica particles, whereas the much more delicate linear worms underwent fragmentation under the same conditions. Similar results were obtained with 4 μm silica particles.

## Abstract 2

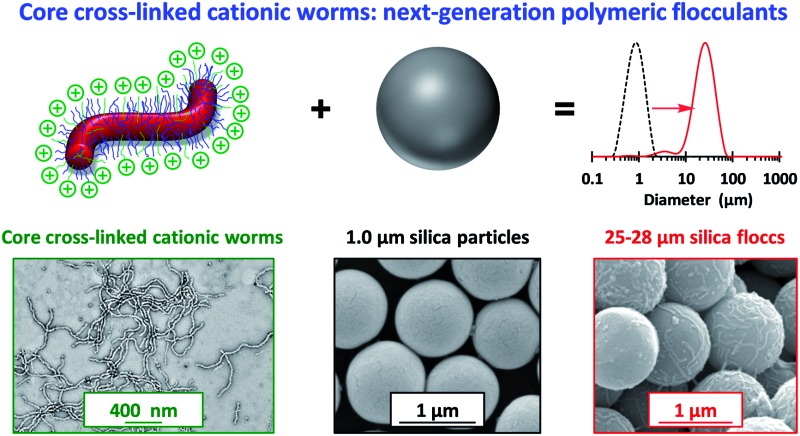
Cationic diblock copolymer worms can be used as flocculants for micrometer-sized silica particles provided that they are covalently stabilized via core cross-linking.

## Introduction

**Scheme 1:**
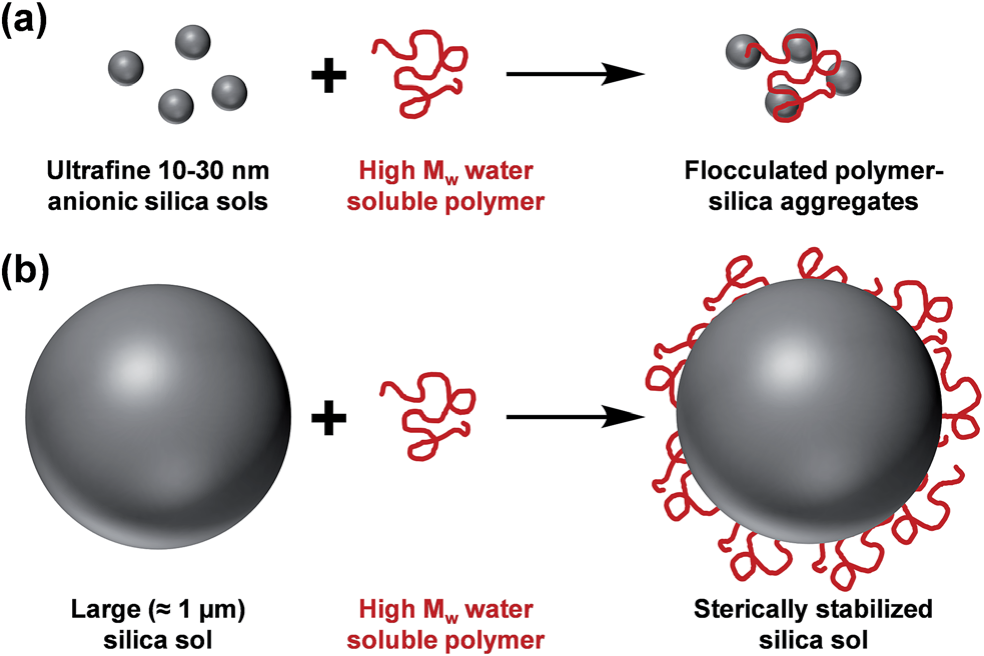
(a) Flocculation of nanometer-sized silica sols and (b) steric stabilization of micrometer-sized silica sols on addition of a high molecular weight water-soluble polymer.

The controlled aggregation of colloidal particles plays a vital role in many important industrial processes such as paper manufacture^1,2^ mineral separation^3^ and water purification.^4–6^ Historically, silica suspensions have been used as models to assess the flocculation efficiency of various high molecular weight water-soluble polymers.^7,8^ Typically, *soluble* cationic polyelectrolytes (or non-ionic polymers)^9–13^ have been evaluated as flocculants for such anionic particles. This approach is well-established for *nano-sized* silica particles, since the length scales of the particles and the flocculant are comparable (see Scheme 1a). However, for *micrometer-sized* silica particles, this usually leads to steric stabilization rather than bridging flocculation (see Scheme 1b). This qualitatively different behavior is the result of the mismatch in length scales for the two components. However, as far as we are aware, polyelectrolytic block copolymer *nanoparticles* have not yet been evaluated as flocculants for relatively large silica particles. In particular, *cylindrical* or *worm-like* nanoparticles can be formed by various diblock copolymers for a relatively narrow range of compositions.^14–25^ The highly anisotropic nature of such nanoparticles leads to a much longer effective length scale, which should enable effective inter-particle bridging. More specifically, in the present study we hypothesized that *cationic worms* might act as efficient flocculants.

Over the last decade or so, various research groups have demonstrated the versatility of polymerization-induced self-assembly (PISA) for the design of bespoke functional AB diblock copolymer nanoparticles.^26–46^ Such PISA formulations are based on either dispersion or emulsion polymerization and can be conducted at relatively high copolymer concentrations (up to 50% w/w) in either polar (*e.g.* water or lower alcohols) or non-polar solvents (*e.g. n*-alkanes, mineral oil or poly(α-olefins)).^47–53^ Briefly, a macromolecular chain transfer agent (macro-CTA) is used as a soluble stabilizer ‘A’ block and self-assembly occurs *in situ* as the growing second ‘B’ block gradually becomes insoluble in the polymerization medium. Various nanoparticle morphologies can be accessed using this approach, including spheres, worms, unilamellar vesicles, oligolamellar vesicles, framboidal vesicles and platelet-like lamella sheets.^54–57^ In particular, the worm morphology has received much recent attention since it offers interesting applications such as sterilisable biocompatible hydrogels,^57^ viscosity modifiers,^19,58^ 3D cell culture media,^59^ efficient Pickering emulsifiers,^60^ a cost-effective storage medium for stem cell transportation and the effective cryopreservation of red blood cells.^61^ Furthermore, certain diblock copolymer worms can undergo an order–order morphology transition on exposure to external stimuli such as pH or temperature.^62–67^ Several techniques have been developed for the preparation of cross-linked block copolymer nanoparticles.^15,19,68–74^ *In situ* core cross-linking of nanoparticles prepared by PISA can be achieved by either (i) addition of a divinyl comonomer during the latter stages of the polymerization^60,75^ or (ii) the post-polymerization addition of a suitable cross-linking agent.^76^ The preparation of cross-linked spheres or vesicles is relatively straightforward.^60,71,77,78^ However, the preparation of cross-linked block copolymer worms is much more challenging. This is in part because of their tendency to form free-standing gels under the PISA synthesis conditions, which makes the post-polymerization addition of cross-linker reagents somewhat problematic.^58^ Moreover, addition of a divinyl comonomer can sometimes lead to (partial) loss of the desired worm copolymer morphology, because this occupies relatively narrow phase space.^60^ Nevertheless, Lovett and co-workers have recently reported the preparation of core cross-linked worms *via* statistical copolymerization of 2-hydroxypropyl methacrylate (HPMA) and glycidyl methacrylate (GlyMA) to form an epoxy-functional core-forming block. Post-polymerization addition of 3-aminopropyl triethoxysilane (APTES) leads to an epoxy-amine reaction within the worm cores, with concomitant siloxane hydrolysis and condensation with secondary hydroxyl groups located on neighboring HPMA residues leading to extensive cross-linking.^76^ Such non-ionic cross-linked worms remained colloidally stable in the presence of excess methanol (which is a good solvent for the core-forming block) or on addition of anionic surfactant.

Semsarilar and co-workers reported that using polyelectrolytic macro-CTAs in PISA formulations typically leads to purely spherical morphologies due to the strong lateral repulsion between the charged stabilizer chains.^79,80^ Even with the addition of salt to screen the unfavorable electrostatics, higher order morphologies such as worms and vesicles could not be observed. However, judicious dilution of the polyelectrolytic stabilizer blocks *via* addition of a non-ionic macro-CTA during the PISA synthesis allowed access to both worms and vesicles.^80^ Unfortunately, such *linear* anionic or cationic worms rapidly dissociate to form individual copolymer chains in the presence of surfactant. Moreover, negative zeta potentials were observed above pH 7 as a result of the relatively short cationic poly([2-(methacryloyloxy)ethyl]trimethylammonium chloride) (PQDMA_32_) macro-CTA utilized, although the use of a carboxylic acid-based RAFT CTA and azo initiator in this PISA formulation may also have contributed to this problem.

Herein we report the synthesis of both linear and cross-linked cationic block copolymer worms using a binary macro-CTA approach *via* RAFT-mediated PISA. The two macro-CTAs employed in this approach are poly(ethylene oxide) (PEO) and PQDMA. The colloidal stability of the resulting nano-objects in the presence of methanol or excess cationic surfactant is compared using dynamic light scattering (DLS) and transmission electron microscopy (TEM). Both types of cationic worms are evaluated as putative flocculants for aqueous dispersions of micrometer-sized silica particles using scanning electron microscopy (SEM) and laser diffraction. A critical comparison of their performance is made with various commercial *soluble* polymeric flocculants.

## Results and discussion

### Synthesis of macromolecular chain transfer agents

The PEO_113_-PETTC macro-CTA used in this work was synthesized as described by Warren and co-workers,^48^ see Scheme S1.† A commercially available poly(ethylene oxide) monomethyl ether precursor (PEO_113_-OH) was modified to give a mesylate adduct that was reacted with ammonia to produce a mono-aminated PEO_113_-NH_2_ (Scheme S1a†). The synthesis of the succinimide ester RAFT agent precursor, SPETTC, has been previously reported by Penfold and co-workers.^62^ The mono-aminated PEO_113_-NH_2_ was reacted with SPETTC to produce the desired PEO_113_-PETTC macro-CTA. ^1^H NMR and UV spectroscopy analysis indicated an end-group functionality of 96% and 94%, respectively (see Fig. S1 in the ESI†). THF gel permeation chromatography (GPC) analysis indicated a *M*_n_ of 4400 g mol^–1^ and a *M*_w_/*M*_n_ = 1.08 *vs.* PEO standards (see Fig. S2†). 2-(Methacryloyloxy)ethyl trimethylammonium chloride (QDMA) was selected as the polyelectrolytic monomer in view of its pH-independent cationic character and commercial availability. A kinetic study of the RAFT aqueous solution polymerization of QDMA at 44 °C using MPETTC was undertaken at pH 4 (see Scheme S1†). These conditions were selected to ensure protonation of the morpholine end-group of this RAFT agent and hence ensure its aqueous solubility.^62^ A mean degree of polymerization (DP) of 120 was targeted at 30% w/w solids. Fig. S3a† shows the monomer conversion *vs.* time curve and corresponding semi-logarithmic plot, while Fig. S3b† shows the evolution in number-average molecular weight, *M*_n_ and dispersity (*M*_w_/*M*_n_) with monomer conversion. After a brief induction period of around 10 min, the polymerization proceeded at a relatively fast rate. More than 99% QDMA conversion was obtained after 3 h. The linear evolution of molecular weight with monomer conversion indicated that this polymerization has pseudo-living character and proceeded under good RAFT control, as expected. *M*_w_/*M*_n_ values are reduced from 1.32 to less than 1.25 during the polymerization. The non-zero *y*-intercept of 12.5 kg mol^–1^ is an experimental artifact that is attributed to inadequate resolution in the low molecular weight limit as a result of overlap between the polymer signal and low molecular species (monomer and/or CTA). A RAFT agent efficiency of 86% was estimated using ^1^H NMR spectroscopy by comparing the theoretical target PQDMA DP with the experimental DP obtained at the end of the kinetic study (after allowing for the final conversion). Under identical conditions, a large batch of PQDMA_125_ macro-CTA with *M*_n_ = 31 800 g mol^–1^ and *M*_w_/*M*_n_ = 1.19 (Fig. S2†) was prepared in order to enable a detailed phase diagram to be constructed, along with the other experiments. The PQDMA DP was calculated by comparing the integrated aromatic signals assigned to the RAFT end-group at 7.2–7.4 ppm to those due to the methacrylic backbone at 0.8–2.4 ppm (Fig. S4†).

### Construction of ([1 – *n*]PEO_113_ + *n*PQDMA_125_)-PHPMA_*y*_ phase diagram

**Scheme 2:**
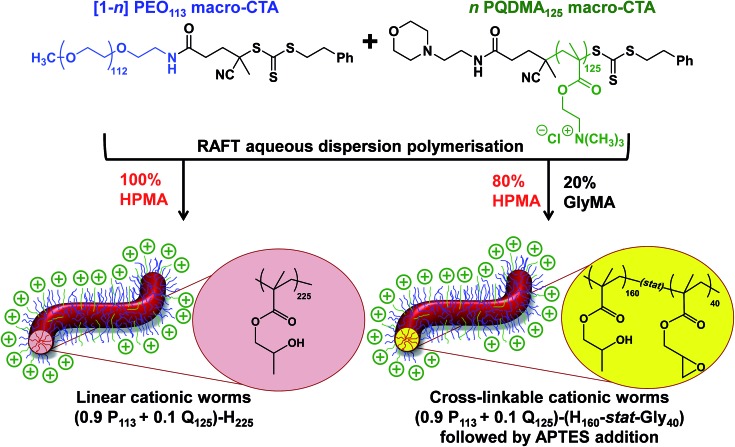
The synthesis of either linear or core cross-linked cationic diblock copolymer worms was achieved *via* RAFT aqueous dispersion homopolymerization of 2-hydroxypropyl methacrylate (HPMA) or statistical copolymerization of HPMA with glycidyl methacrylate (GlyMA) using a binary mixture of poly(ethylene oxide) and poly(2-(methacryloyloxy)ethyl trimethylammonium chloride) (PQDMA) chain transfer agents. Here *n* represents the mole fraction of PQDMA_125_ in the binary mixture of PQDMA_125_ and PEO_113_ macro-CTAs. For brevity, H and Gly denote HPMA and GlyMA, respectively and P and Q refer to the PEO and PQDMA stabilizer blocks.

**Fig. 1:**
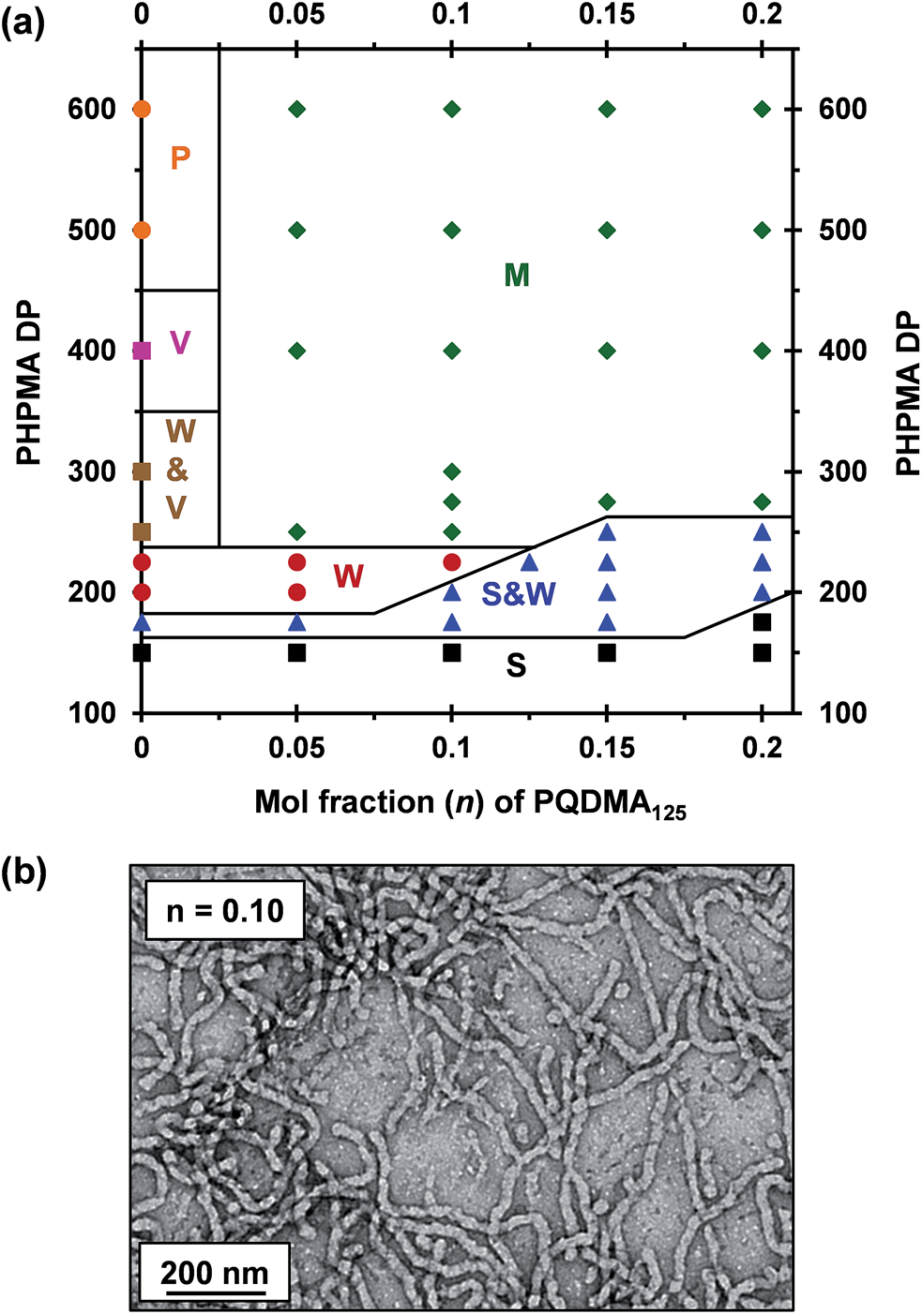
(a) Phase diagram constructed for the RAFT aqueous dispersion polymerization of HPMA at a fixed copolymer concentration of 20% w/w solids at 50 °C using a binary mixture of PEO_113_-PETTC and PQDMA_125_ macro-CTAs [S = spheres, W = worms, V = vesicles, M = mixed phase and P = precipitate]. (b) Representative TEM image for linear cationic (0.9PEO_113_ + 0.1PQDMA_125_)-PHPMA_225_ worms.

A binary mixture of PEO_113_ and PQDMA_125_ macro-CTAs was chain-extended with HPMA under RAFT aqueous dispersion polymerization conditions to produce linear cationic diblock copolymer nano-objects, see Scheme 2. A phase diagram was constructed at a constant copolymer concentration of 20% w/w solids (see Fig. 1) whereby the mol fraction (*n*) of the PQDMA_125_ macro-CTA was systematically varied from 0 to 0.20 while targeting DPs of 150 to 600 for the PHPMA core-forming block. For all syntheses, the final HPMA conversion exceeded 99% and the final copolymer morphology was assigned by TEM studies. The general formula for this series of block copolymer nanoparticles is given by ([1 – *n*]PEO_113_ + *n*PQDMA_125_)-PHPMA_*z*_, where *n* is the mol fraction of PQDMA_125_ and *z* is the target DP of the PHPMA block. Unfortunately, these linear cationic nanoparticles cannot be analyzed by GPC because there is no suitable eluent that dissolves all three blocks (*i.e.* hydrophilic PEO_113_ and PQDMA_125_ plus the hydrophobic PHPMA).

In separate experiments, both PEO_113_ and PQDMA_125_ macro-CTAs were chain-extended to assess their blocking efficiency. PEO_113_ was chain-extended with 250 units of HPMA, and in this case the resulting diblock copolymer is amenable to GPC analysis.^48^ In contrast, a self-blocking experiment was performed with the PQDMA_125_ macro-CTA using 350 units of QDMA to target an overall DP of 475. THF and aqueous GPC analysis indicated high blocking efficiencies for both the PEO_113_ and PQDMA_125_ macro-CTAs (see Fig. S2†).

To examine the effect of conferring cationic character on the diblock copolymer nanoparticles, a series of PEO_113_-PHPMA_*z*_ diblock copolymer PISA syntheses were performed as control experiments. A pure sphere phase was obtained when targeting PEO_113_-PHPMA_150_ while a mixed phase of spheres and worms was identified for PEO_113_-PHPMA_175_, which is in good agreement with previous work by Warren *et al.* ^48^ Free-standing worm gels were observed for PHPMA DPs of 220 to 225 (see Fig. 1), with a mixed phase of worms and vesicles being observed for PHPMA DPs of 250 to 300. At a PHPMA DP of 400, a pure vesicle phase was identified, but precipitation occurred when targeting a DP of 500. Addition of PQDMA_125_ (*n* = 0.05) to such PISA syntheses has no discernible effect on the phase diagram when targeting PHPMA DPs of 225 or below. However, the worm/vesicle binary mixed phase and pure vesicle phase are no longer observed above this critical PHPMA DP. Instead, only rather ill-defined copolymer morphologies are obtained, such as mixed phases of worms and lamella-like sheets or mixtures of spheres, vesicles and tubular vesicles (see Fig. S5†). However, precipitation does not occur at a PHPMA DP of 500 or 600 when PQDMA_125_ is incorporated as a supplementary stabilizer block. Presumably, the polyelectrolytic character of this macro-CTA boosts the steric stabilization conferred by the non-ionic PEO_113_, thus facilitating the formation of colloidally stable nano-objects (see Fig. S5†). Increasing the PQDMA_125_ mol fraction (from *n* = 0.05 to *n* = 0.10) in this PISA formulation has a relatively modest effect on the phase diagram. The only discernible change is at a PHPMA DP of 200, where a sphere/worm mixed phase is observed, indicating narrowing of the worm phase space. A representative TEM image of linear (0.9PEO_113_ + 0.1PQDMA_125_)-PHPMA_225_ worms is shown in Fig. 1b. A pure worm phase is no longer observed on increasing *n* up to 0.125.

**Fig. 2:**
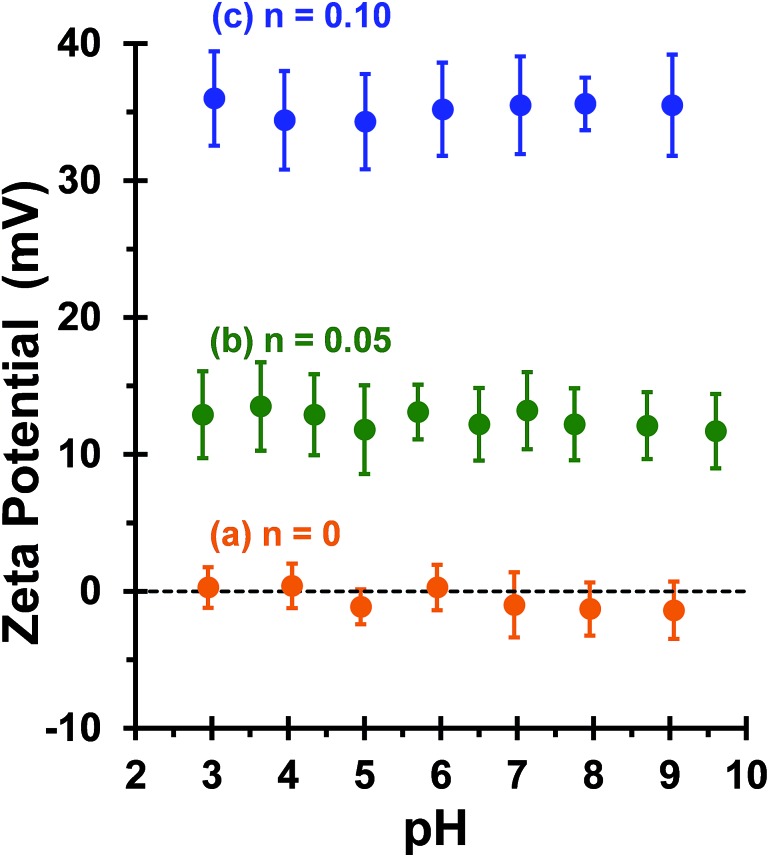
Zeta potential *vs.* pH curves obtained for a series of ([1 – *n*]PEO_113_ + *n*PQDMA_125_)-PHPMA_225_ linear diblock copolymer worms prepared at 20% w/w solids by the RAFT aqueous dispersion polymerization of HPMA at 50 °C, where (a) *n* = 0, (b) *n* = 0.05 and (c) *n* = 0.10. Zeta potentials were determined at 20 °C for 0.1% w/w copolymer dispersions in the presence of 1 mM KCl. Error bars are equivalent to one standard deviation. The aqueous dispersion pH was adjusted using either 0.1 M or 1 M HCl.

This is consistent with recent work by Williams and co-workers,^81^ who utilized poly(glycerol monomethacrylate) (PGMA) instead of PEO_113_ as a non-ionic stabilizer block in combination with a PQDMA_95_ macro-CTA to produce cationic thermoresponsive worm gels with weak anti-microbial activity. Thus, if a pure worm phase is desired, the maximum proportion of PQDMA_125_ that can be incorporated into the present PISA formulation is *n* = 0.10. This constraint arises because the worm phase is relatively narrow, as reported previously.^82^ Aqueous electrophoresis was used to characterize the cationic character of three pure diblock copolymer worms at a fixed PHPMA DP of 225 (see Fig. 2). As expected, the PEO_113_-PHPMA_225_ copolymer worm control exhibited a zeta potential of approximately zero across the entire pH range studied. Introduction of PQDMA_125_ into the stabilizer block (*n* = 0.05) led to initially weak cationic character, as indicated by a pH-independent zeta potential of approximately +13 mV. However, a relatively high zeta potential of +35 mV was observed on doubling the mol fraction of PQDMA_125_ (*n* = 0.10) and again there was no discernible change over a wide pH range. In all cases, simultaneous DLS studies confirmed that there was no change in the ‘sphere-equivalent’ hydrodynamic diameter on varying the solution pH, suggesting that the original worm morphology was retained during these electrophoresis studies.

### Covalent cross-linking and colloidal stability of cationic diblock copolymer worms

Lovett *et al.* reported that 3-aminopropyl triethoxysilane (APTES) can be used to cross-link epoxy-functionalized worms *via* a post-polymerization protocol.^76^ Worm core cross-linking involves reaction of the epoxy groups on the GlyMA residues with APTES, with concomitant hydrolysis to form silanol groups that condense with other silanol groups and/or secondary hydroxyl groups located on neighboring HPMA residues (see Scheme S2†). These reactions lead to extensive cross-linking within the worm cores. ^1^H NMR was used to monitor this complex process and it was found that epoxide ring-opening and hydrolysis/condensation occurred on comparable time scales.^76^ Such covalently-stabilized non-ionic worms remained colloidally stable in the presence of either methanol or anionic surfactant, whereas the linear precursor worms underwent rapid dissociation under the same conditions. An increase in storage modulus (*G*′) was observed after core cross-linking, which is presumably the result of an increase in the worm persistence length.^76^

**Fig. 3:**
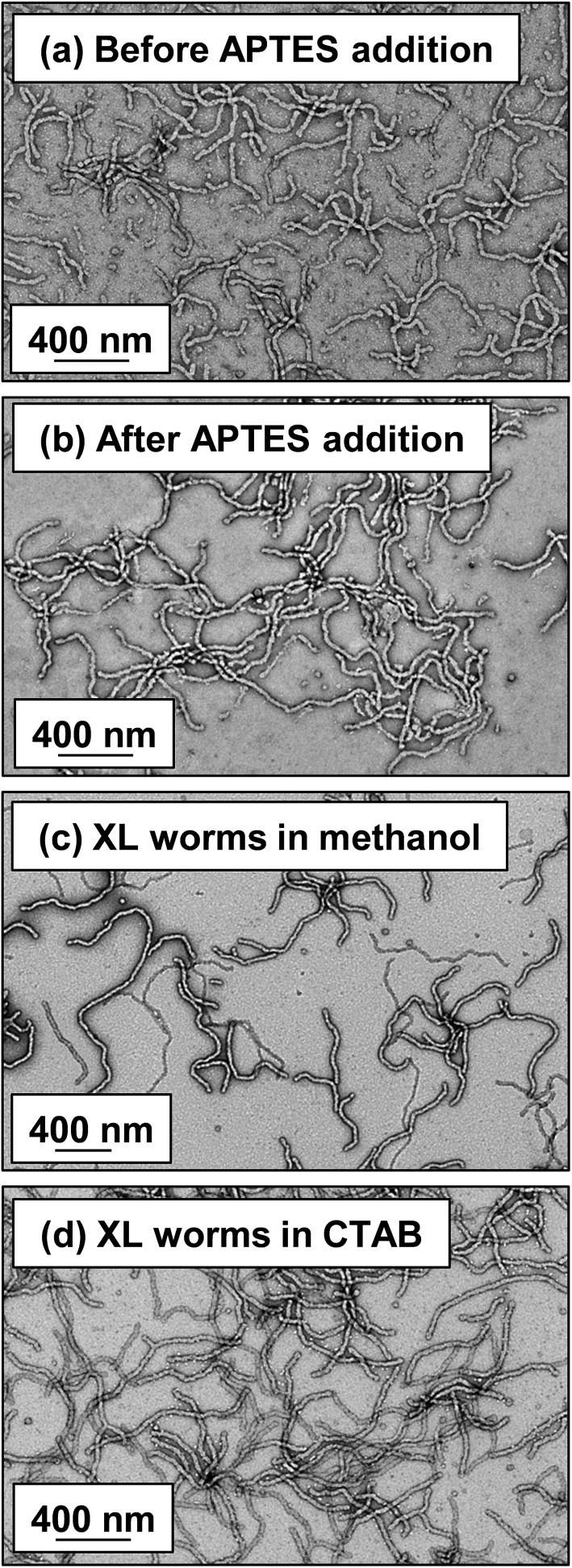
Representative TEM images recorded for 0.1% w/w copolymer worm dispersions dried at pH 9: (a) (0.9PEO_113_ + 0.1PQDMA_125_)-P(HPMA_160_-*stat*-GlyMA_40_) worms pre-APTES addition, (b) core cross-linked (0.9PEO_113_ + 0.1PQDMA_125_)-P(HPMA_160_-*stat*-GlyMA_40_) worms after APTES addition, and the same copolymer worms in the presence of (c) 0.1% w/w CTAB and (d) diluted from 7.5% w/w copolymer to 0.1% w/w copolymer using methanol [abbreviations: XL = core cross-linked and CTAB = cetyltrimethylammonium bromide].

In view of these prior observations, we decided to examine the PISA synthesis of core cross-linked cationic diblock copolymer worms and assess their colloidal stability in the presence of either methanol or a well-known cationic surfactant, cetyltrimethylammonium bromide (CTAB). The mol fraction, *n*, of PQDMA_125_ was fixed at 0.10 in order to maximize the cationic character of the copolymer nanoparticles while maintaining a pure worm phase. The PHPMA core-forming block was replaced with a statistical copolymer comprising 80 mol% HPMA and 20 mol% GlyMA. However, introducing the GlyMA comonomer led to a subtle change in the phase diagram, with a mixed phase of worms and vesicles being observed instead of the desired pure worm phase (Fig. S6†). Thus the overall DP of the core-forming block was adjusted from 225 to 200 to compensate for the presence of the GlyMA comonomer. A very high comonomer conversion (>99%) was achieved to afford well-defined linear (0.9PEO_113_ + 0.1PQDMA_125_)-P(HPMA_160_-*stat*-GlyMA_40_) diblock copolymer worms. Unfortunately, PISA synthesis at 20% w/w solids produced a rather strong copolymer gel, which made APTES dissolution for post-polymerization cross-linking somewhat problematic. Hence this worm gel was diluted to 7.5% w/w solids using deionized water prior to APTES addition, followed by gentle stirring for 24 h at room temperature. TEM studies confirmed the presence of both linear (0.9PEO_113_ + 0.1PQDMA_125_)-P(HPMA_160_-*stat*-GlyMA_40_) diblock copolymer worms and cross-linked (0.9PEO_113_ + 0.1PQDMA_125_)-P(HPMA_160_-*stat*-GlyMA_40_) diblock copolymer worms (see Fig. 3). Chambon and co-workers^71^ reported using DLS and TEM to assess the colloidal stability of block copolymer vesicles in the presence of various aqueous surfactant solutions. Herein we utilize the same approach to examine the colloidal stability of both linear and core cross-linked cationic worms in the presence of either methanol (which is a good solvent for the core-forming block) or 0.1% w/w CTAB. A 0.1% w/w CTAB solution corresponds to a concentration of 2.7 mM, which is above the critical micelle concentration for CTAB reported in the literature.^83,84^ Table S1† summarizes the intensity-average diameters, zeta potentials and the derived count rates obtained for linear and cross-linked cationic worms (i) dispersed in mildly alkaline aqueous solution (pH 9), (ii) in the presence of 0.1% w/w CTAB (also at pH 9), or (iii) as a methanolic dispersion. It is important to note that the ‘sphere-equivalent’ hydrodynamic diameter reported by DLS does not correspond to either the mean worm length or the mean worm width. Notwithstanding this limitation, this sizing technique suggests that the dimensions of the linear and cross-linked worms at pH 9 are comparable.

Furthermore, mean zeta potentials obtained for the cross-linked and linear worms are very similar (approximately +35 mV). The linear worm dispersion diluted in methanol has a very low normalized light scattering intensity, which suggests worm dissociation under these conditions. In contrast, the relatively high light scattering intensity observed for cross-linked worms in the same solvent indicates that the original vermicious morphology is preserved under these conditions. The slightly higher ‘sphere-equivalent’ diameter for the cross-linked worms in methanol compared to the same worms in water (240 nm *vs.* 216 nm) most likely indicates some degree of worm swelling, which would also account for the modest (*ca.* 10%) reduction in the light scattering intensity. Thus successful cross-linking of the worm cores prevents molecular dissolution occurring under these conditions. It is also instructive to compare the linear and cross-linked worms exposed to the presence of 0.1% w/w CTAB. The relatively low normalized intensity observed for the former dispersion suggests near-molecular dissolution of the linear worms, whereas the cross-linked worms clearly survive the CTAB challenge. Indeed, TEM studies of the corresponding dried dispersions confirm that the cross-linked worms survive exposure to 0.1% w/w CTAB or dilution from 20% w/w to 0.1% w/w solids using methanol co-solvent (see Fig. 3).

### Flocculation of micrometer-sized silica particles

Bridging flocculation typically involves the adsorption of a high molecular weight water-soluble polymer onto two or more relatively small colloidal nanoparticles, which promotes their aggregation. For example, Solberg and co-workers reported using high molecular weight polyacrylamide for the flocculation of 20 nm aqueous silica sols.^8^ Other well-known flocculants include high molecular weight poly(ethylene oxide) or poly(*N*-vinylpyrrolidone).^85,86^ Similarly, Mabire *et al.* found that cationic polyelectrolytes can act as highly effective flocculants for 125 nm anionic silica particles.^87^ However, for the flocculation of much larger (micrometer-sized) particles, the bridging flocculation mechanism is likely to fail. This is because the markedly different length scales between the particles and the soluble polymer chains favour steric stabilization (*i.e.* the soluble polymer adsorbs onto and fully coats individual particles, see Scheme 1). In the present study, both linear and cross-linked cationic worms were evaluated as putative flocculants for micrometer-sized silica particles, with various high molecular weight water-soluble polymers being used as negative controls.

**Fig. 4:**
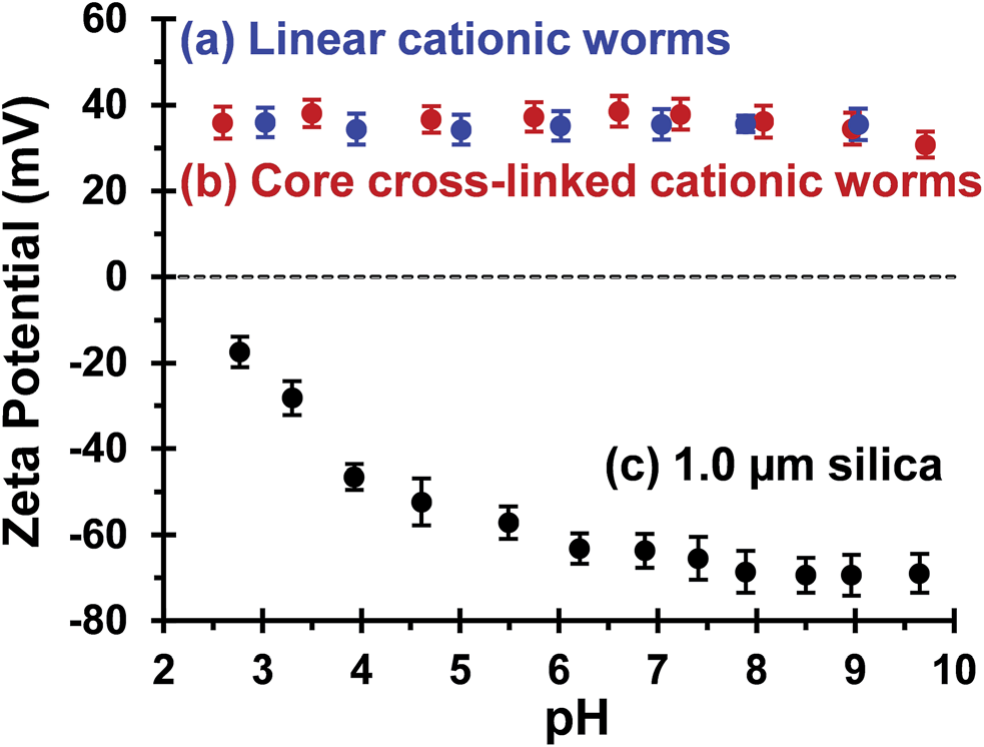
Zeta potential *vs.* pH curves recorded for (a) linear (0.9PEO_113_ + 0.1PQDMA_125_)-PHPMA_225_ worms, (b) core cross-linked (0.9PEO_113_ + 0.1PQDMA_125_)-P(HPMA_160_-*stat*-GlyMA_40_) worms and (c) 1.0 μm silica particles. The linear worm data set is taken from Fig. 2 to enable direct comparison. Measurements were conducted at 20 °C on 0.1% w/w dispersions in the presence of 1 mM background KCl. The dispersion pH was adjusted by addition of either 1.0 M or 0.1 M HCl.

In principle, core cross-linking should make the worm morphology much more robust. Moreover, stiffer worms should be obtained with a greater mean persistence length, which should aid worm adsorption onto multiple silica particles. In initial experiments, zeta potential *vs.* pH curves were constructed for 0.1% w/w aqueous dispersions of linear worms, core cross-linked worms and silica particles. The bare silica particles exhibit a volume-average diameter, *D*_[4/3]_ of 1.0 μm, as judged by laser diffraction studies. A zeta potential of –69 mV was observed for these silica particles at pH 9, whereas the linear and cross-linked cationic worms had comparable zeta potentials of +35 mV and +34 mV, respectively (see Fig. 4). As expected, both worm dispersions exhibited pH-independent electrophoretic behaviour, whereas the zeta potential for the 1.0 μm silica particles gradually decreased to –20 mV at pH 2.8. Thus the flocculation study was performed at pH 9 in order to maximize the electrostatic interaction. All flocculation studies were conducted using 1.0% w/w silica. The adsorbed amount (*i.e.* the worm mass per unit surface area of silica) was systematically varied in order to assess the effectiveness of the cationic worms as flocculants for the silica particles. The specific surface area, *A*_s_, of these silica particles can be calculated using *A*_s_ = 3/(*ρ*_silica_ *R*), where *ρ*_silica_ and *R* are the density and mean radius of the silica particles, respectively. The solid-state density of the 1.0 μm diameter silica particles, *ρ*_silica_, was found to be 2.03 g cm^–3^ by helium pycnometry. Using this density, *A*_s_ is estimated to be 2.9 m^2^ g^–1^.

**Fig. 5:**
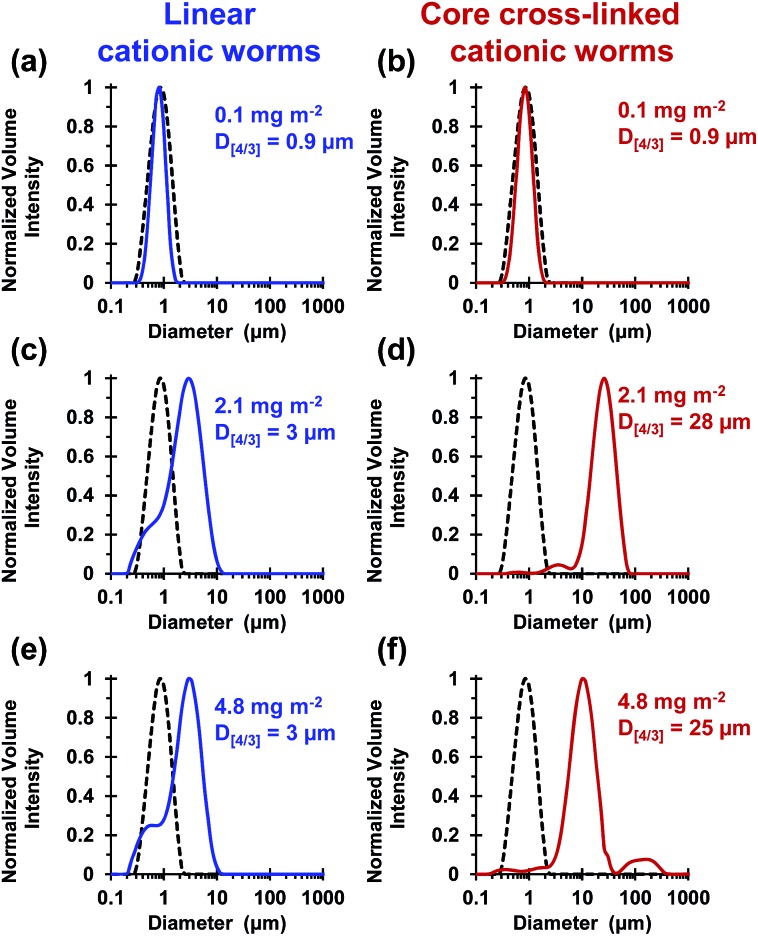
Volume-average particle size distributions obtained *via* laser diffraction for the attempted flocculation of 1.0 μm silica at pH 9 using either linear (0.9PEO_113_ + 0.1PQDMA_125_)-PHPMA_225_ worms (blue traces) or core cross-linked (0.9PEO_113_ + 0.1PQDMA_125_)-P(HPMA_160_-*stat*-GlyMA_40_) worms (red traces) at adsorbed amounts of (a, b) 0.1 mg m^–2^ (c, d) 2.1 mg m^–2^ and (e, f) 4.8 mg m^–2^, respectively. The black dotted traces represent the volume-average particle size distribution obtained for the pristine 1.0 μm silica particles in the absence of any worms.

These silica particles were added to the cross-linked cationic worms at nominal adsorbed amounts of 0.1 mg m^–2^, 2.1 mg m^–2^ and 4.8 mg m^–2^. At 0.1 mg m^–2^, laser diffraction studies indicated no significant change in *D*_[4/3]_ for both the linear and the cross-linked cationic worms, confirming that essentially no flocculation of the 1.0 μm silica particles occurred under these conditions (see Fig. 5a).

**Fig. 6:**
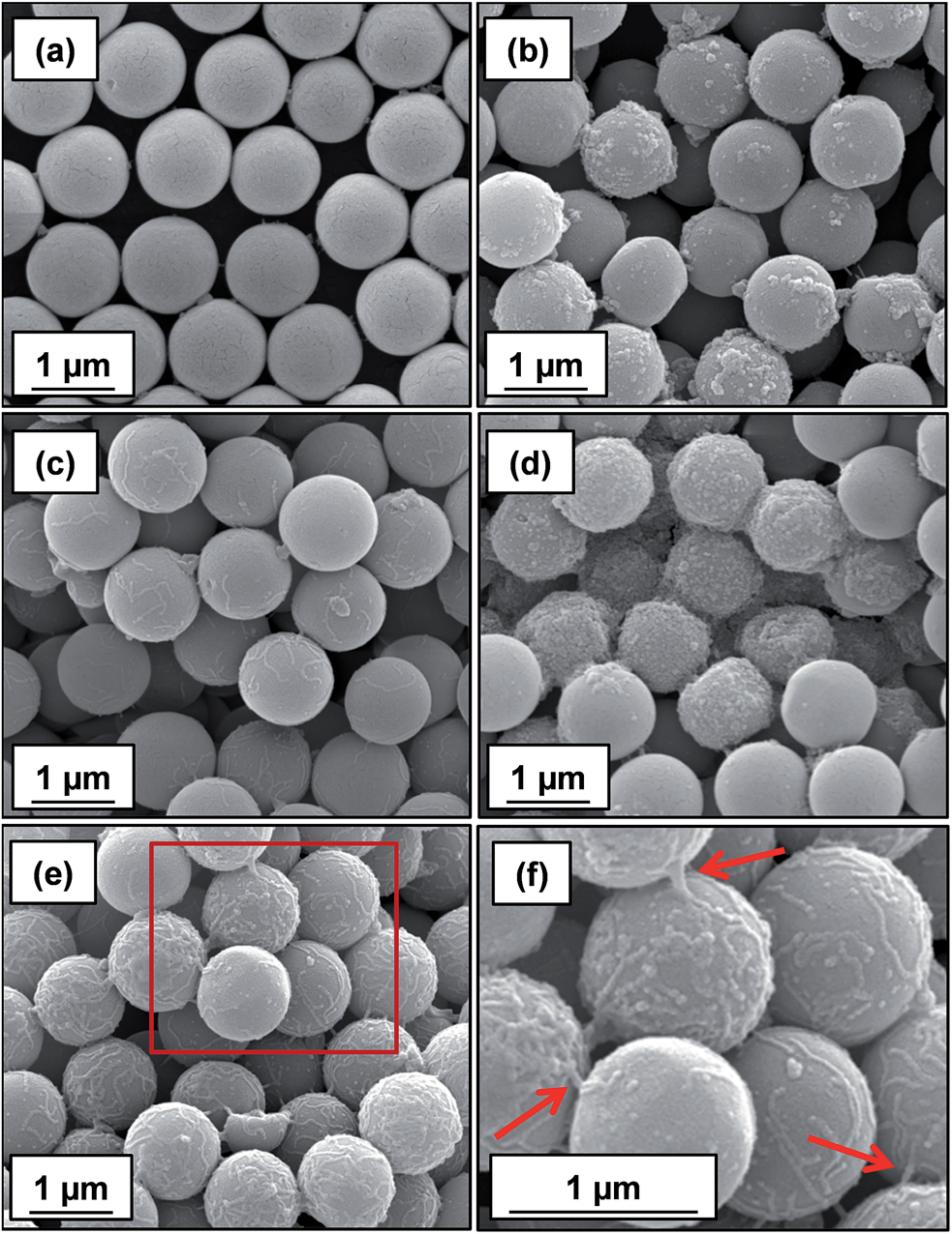
SEM images obtained for (a) bare 1.0 μm silica, (b) silica plus linear cationic worms and (c) silica plus cross-linked cationic worms prepared at a nominal adsorbed amount of 2.1 mg m^–2^, respectively. Images shown in (d) and (e) correspond to 1.0 μm silica particles in the presence of either linear or cross-linked cationic worms at a nominal adsorbed amount of 4.8 mg m^–2^. (f) Magnified image of the area indicated by the red square shown in (e), confirming the presence of cross-linked cationic worms adsorbed intact at the silica particle surface; this provides direct experimental evidence for the bridging flocculation mechanism indicated by laser diffraction studies (see Fig. 5). In each case, worms were adsorbed onto the silica particles ([silica]_0_ = 1.0% w/w) at pH 9 followed by drying at 20 °C overnight prior to SEM inspection.

At a higher nominal adsorbed amount of 2.1 mg m^–2^, laser diffraction indicated a *D*_[4/3]_ of 3 μm for the linear cationic worms, suggesting only rather weak flocculation (Fig. 5c). However, the cross-linked cationic worms act as a highly effective flocculant, with a *D*_[4/3]_ of 28 μm being observed (Fig. 5b). Increasing the nominal adsorbed amount to 4.8 mg m^–2^ confirmed the superior flocculation performance of cross-linked worms compared to that of the linear worms, with *D*_[4/3]_ diameters of 25 μm and 3 μm being observed respectively (compare Fig. 5e and f). SEM studies were conducted on 1.0 μm silica particles before and after exposure to either linear or cross-linked cationic worms. The pristine 1.0 μm silica particles (Fig. 6a) are spherical, uniform in size and have a smooth surface morphology. At a nominal adsorbed amount of 2.1 mg m^–2^, SEM studies provide no evidence for the linear worms surviving electrostatic adsorption onto the silica surface (Fig. 6b). Instead, only relatively small, pseudo-spherical structures can be observed. However, when cross-linked worms are used under the same conditions, intact adsorbed worms are clearly discernible at the silica particle surface (Fig. 6c). Using a nominal adsorbed amount of 4.8 mg m^–2^ leads to similar observations, but higher surface coverage of the silica particles is achieved in each case (compare Fig. 6b with 6d and also Fig. 6c with 6e).

Close inspection of Fig. 6f indicates that some of the cross-linked cationic worms span between adjacent silica particles (see red arrows in Fig. 6f). This provides direct evidence that the particle aggregation observed by laser diffraction is indeed the result of a bridging flocculation mechanism.

Cross-linked worms are much more effective flocculants than linear worms because they are much more robust: covalent stabilization of the worm cores is essential to preserve the original copolymer morphology after electrostatic adsorption of the cationic worms onto the anionic silica particles. In striking contrast, the linear cationic worms break up following their adsorption onto the relatively massive silica particles to form two distinct populations of (mainly) non-ionic PEO_113_-PHPMA_225_ and (mainly) cationic PQDMA_125_-PHPMA_225_ nanoparticles, with each possessing a pseudo-spherical morphology (see Scheme S3†). The hydrophobic nature of the core-forming PHPMA block drives formation of the linear worms during PISA. However, this weak physical interaction is clearly insufficient to maintain the original morphology once these cationic worms adsorb onto the anionic silica particles.

Image J software was used to assess worm dimensions from TEM images. Analysis of 50 worms indicated a mean worm length, *L*_w_, of 956 nm and a mean worm radius, *r*_w_, of 15 nm. If the worm morphology is approximated to that a cylinder of volume *V* (where *V* = π*r*_w_2*L*_w_) and taking the worm density, *ρ*_w_, to be that of the PHPMA core-forming block (1.15 g cm^–3^), we estimate the mean mass, *m*, (where *m* = *ρ*_w_ *V*) of a single worm to be 7.77 × 10^–16^ g. Note that the mass, *M*, of a single 1.0 μm silica particle, using *M* = *ρ*_silica_ × 4/3π*R* ^3^ (where *R* is the silica particle radius), is calculated to be 1.12 × 10^–12^ g, which is approximately 1450 times greater than that of a single worm. Thus the linear cationic worms are simply unable to survive the strong torsional forces exerted on them by the much more massive silica particles during Brownian motion. Electrostatic interactions lead to strong adsorption of the cationic worms onto the anionic silica particles, so the failure mechanism involves disruption of the physical van der Waals forces between the weakly hydrophobic PHPMA chains within the worm cores. In order to gain further mechanistic insight, an aqueous dispersion comprising a binary mixture of 1.0 μm silica particles plus linear copolymer worms prepared at a nominal adsorbed amount of 4.8 mg m^–2^ was centrifuged at 6000 rpm for 1 h. After careful removal of the aqueous supernatant, the sedimented silica particles were redispersed in water at pH 9. Aqueous electrophoresis studies conducted at pH 9 indicated a zeta potential of only –17 mV, which is significantly lower than that of the original silica particles (see Fig. 4). This suggests that the pseudo-spherical particles that remain on the surface of the silica particles (see Fig. 6d) comprise mainly cationic PQDMA_125_-PHPMA_225_ chains. According to Semsarilar *et al.*, copolymer nanoparticles comprising mainly PQDMA_125_-PHPMA_225_ chains would be expected to form spheres, rather than worms.^80^ In addition, adsorption of non-ionic PEO_113_-PHPMA_225_ nanoparticles at the silica surface may also occur. However, DLS studies of the aqueous supernatant solution obtained after sedimentation of the silica particles indicated a mean hydrodynamic particle diameter of 85 nm (DLS polydispersity = 0.19), with aqueous electrophoresis studies indicating a weakly negative zeta potential of –7 mV. TEM studies of this dried supernatant confirmed a pseudo-spherical morphology (Fig. S7†). Based on findings reported by Warren and co-workers, PEO_113_-PHPMA_225_ was expected to self-assemble to form worms in aqueous solution.^48^ Inspecting Fig. 1, this is indeed the case. However, for the highly dilute copolymer concentrations utilized in these flocculation studies, multiple sphere–sphere fusion (which is the critical first step for worm formation^88^) cannot occur, which leads to a kinetically-trapped spherical morphology (see Fig. 1). Thus there is strong experimental evidence to support the *in situ* disintegration of the linear cationic worms during their adsorption onto micrometer-sized silica particles, as summarized in Scheme S3.† It is emphasized that this mechanism does not apply to the cross-linked cationic worms, since covalent stabilization is sufficient to enable their survival after adsorption onto the relatively massive silica particles. This accounts for the marked difference in performance for these two putative bridging flocculants.

**Fig. 7:**
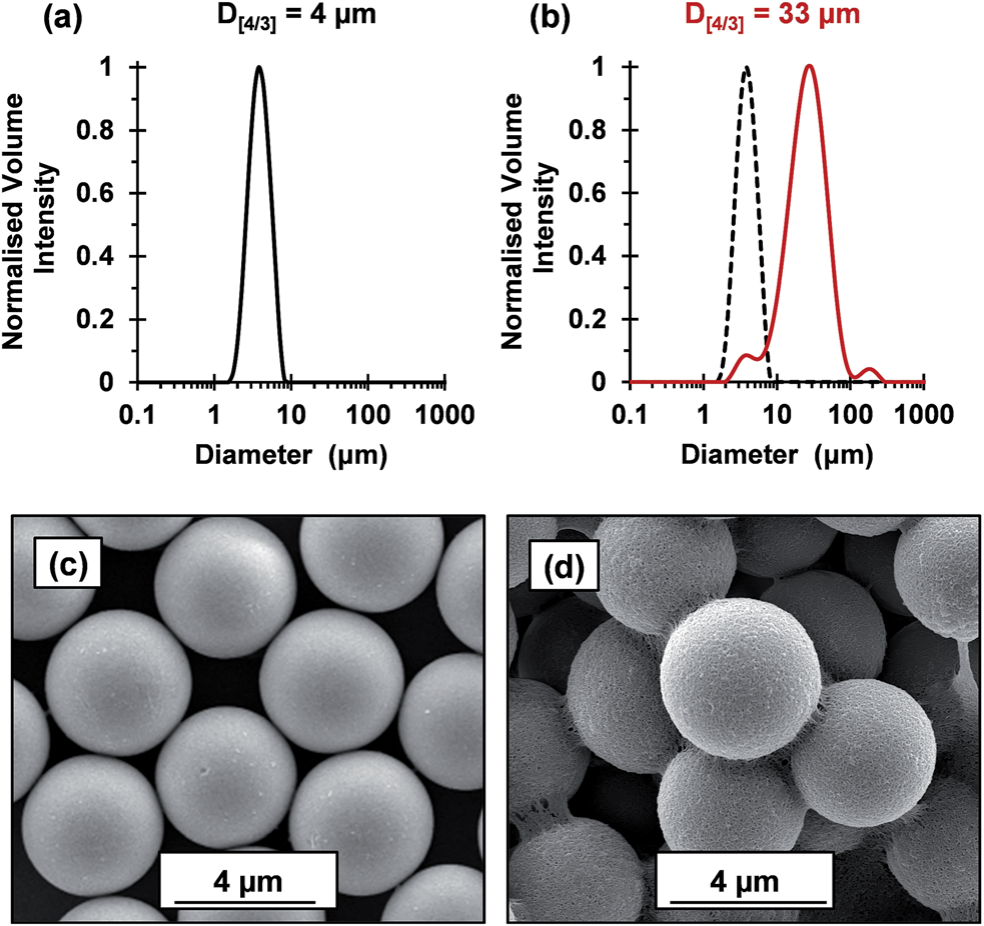
Volume-average particle size distributions obtained *via* laser diffraction for (a) pristine 4 μm silica particles and (b) (0.9PEO_113_ + 0.1PQDMA_125_)-P(HPMA_160_-*stat*-GlyMA_40_) cross-linked worms adsorbed onto 4 μm silica particles at a nominal adsorbed amount of 88 mg m^–2^. Representative SEM images are shown for (c) pristine 4 μm silica particles and (d) the above cross-linked worms adsorbed onto these silica particles, which supports the suggested bridging flocculation mechanism.

The flocculation performance of the cross-linked cationic worms was further examined by attempting flocculation of 4 μm silica particles at pH 9. At this pH, the silica spheres exhibit a zeta potential of –74 mV. Given that the *A*_s_ of these silica spheres is 0.72 m^2^ g^–1^, the silica concentration was increased to 4.0% w/w to maintain a constant silica surface area. No flocculation was observed when using nominal adsorbed amounts of 2.1 mg m^–2^ and 4.8 mg m^–2^, which had been sufficient to flocculate the 1.0 μm silica particles. Thus, this parameter was increased to 88 mg m^–2^ (Fig. 7). Laser diffraction studies confirmed an increase in apparent volume-average diameter from 4 μm for the original silica particles up to 33 μm in the presence of the cross-linked cationic worms. SEM studies indicated that these worms adsorb intact at the silica surface with relatively high surface coverage and readily identifiable worm bridges between adjacent silica particles. It is perhaps noteworthy that the 4.0 μm silica particles were normally added to the cross-linked cationic worms. However, similarly strong flocculation was also observed if this order of addition was reversed (see Fig. S8†). Comparable results were also obtained when using the 1.0 μm silica particles (data not shown). Moreover, such cross-linked cationic worms were also able to flocculate 8 μm silica particles (data not shown).

For comparative purposes, four high molecular weight commercial water-soluble polymers were examined as potential flocculants for the 1.0 μm silica particles at pH 9. These polymers were poly(ethylene oxide) (PEO; *M*_w_ = 4 000 000 g mol^–1^), polyacrylamide (PA; *M*_w_ = 6 000 000 g mol^–1^), poly(*N*-vinylpyrrolidone) (PVP; *M*_w_ = 1 300 000 g mol^–1^) and poly(diallyldimethylammonium chloride) (PDADMAC; *M*_w_ = 500 000 g mol^–1^), see Table 1. The apparent volume-average particle diameters of the silica particles obtained after addition of each of these four commercial polymers to 1.0 μm silica particles at pH 9 at nominal adsorbed amounts of 2.1, 4.8, 17.2 or 34.3 mg m^–2^ are also summarized in Table 1. Little or no flocculation was observed in all cases. Laser diffraction size distributions are either unimodal or bimodal, with peaks at 1.0 μm and approximately 4 μm being observed (see Figs. S9–S12†). However, when the same polymers were added in turn to a 31 nm anionic silica sol ([silica]_0_ = 0.05% w/w), then flocculation was observed in all cases (Table S2, Fig. S13†). In this case the length scales of the silica nanoparticles and the polymer coils are similar (tens of nm). These control experiments serve to illustrate the difficulty of aggregating micrometer-sized particles using conventional water-soluble polymeric flocculants. This highlights the exceptional performance of the cross-linked cationic worms revealed in this study: the mean contour length of these highly anisotropic particles is comparable to the mean silica diameter, which accounts for their ‘superflocculant’ behavior.

**Table 1:** Summary of the volume-average particle diameters obtained *via* laser diffraction after the attempted flocculation of a 1.0% w/w aqueous dispersion of 1.0 μm silica particles at pH 9 using four commercially available water-soluble polymers as putative flocculants

Commercial polymer	*M*_w_ (g mol^–1^)	Volume-average diameter (μm) *via* laser diffraction using various adsorbed amounts of polymer per unit area of silica			
		2.1 mg m^–2^	4.8 mg m^–2^	17.2 mg m^–2^	34.3 mg m^–2^
Poly(ethylene oxide)	4 000 000	2	3	3	3
Polyacrylamide	6 000 000	1	1	1	3
Poly(*N*-vinylpyrrolidone)	1 300 000	2	2	2	3
Poly(diallyldimethylammonium chloride)	500 000	1	1	1	3

## Conclusions

In summary, chain extension of a binary mixture of PEO_113_ and PQDMA_125_ macro-CTAs with HPMA using RAFT aqueous dispersion polymerization at 20% w/w solids can be used to prepare cationic diblock copolymer nano-objects. In particular, incorporation of 10 mol% PQDMA_125_ while targeting an appropriate degree of polymerization for the core-forming PHPMA block enables the formation of linear copolymer worms (zeta potential = +35 mV) that remain highly cationic across a wide pH range. Core cross-linked cationic worms were readily prepared using epoxy-amine chemistry *via* statistical copolymerization of 20% mol GlyMA with HPMA, followed by addition of APTES. Extensive cross-linking occurs *via* reaction of the hydrolyzed pendent silanol groups with the secondary alcohol groups on the HPMA residues. Unlike the corresponding linear worms, these core cross-linked cationic worms can withstand the presence of either a cationic surfactant or methanol. Importantly, such cross-linked cationic worms are much more effective flocculants of highly anionic 1.0 μm silica particles at pH 9. In contrast, the linear cationic worms are much less effective flocculants, because they break up to form a mixture of (mainly) non-ionic and cationic pseudo-spherical block copolymer nanoparticles. To benchmark the exceptional performance of the cross-linked cationic worms, a series of four high molecular weight commercial water-soluble polymers were also evaluated under the same conditions and found to be only weak flocculants for 1.0 μm silica particles. Finally, preliminary experiments confirmed that these cross-linked cationic worms can also flocculate 4 μm (and even 8 μm) anionic silica particles at pH 9.

## References

[cit1] Swerin A., Wågberg L. (1994). Nord. Pulp Pap. Res. J..

[cit2] Nicu R., Bobu E., Desbrieres J. (2011). Cellul. Chem. Technol..

[cit3] Nasser M. S., James A. E. (2006). Sep. Purif. Technol..

[cit4] Kam S.-K., Gregory J. (2001). Water Res..

[cit5] Bolto B., Gregory J. (2007). Water Res..

[cit6] Isik M., Fernandes A. M., Vijayakrishna K., Paulis M., Mecerreyes D. (2016). Polym. Chem..

[cit7] Bleier A., Goddard E. D. (1980). Colloids Surf..

[cit8] Solberg D., Wågberg L. (2003). Colloids Surf., A.

[cit9] Zhou Y., Gan Y., Wanless E. J., Jameson G. J., Franks G. V. (2008). Langmuir.

[cit10] Zhou Y., Franks G. V. (2006). Langmuir.

[cit11] Mende M., Schwarz S., Petzold G., Jaeger W. (2007). J. Appl. Polym. Sci..

[cit12] Flood C., Cosgrove T., Espidel Y., Howell I., Revell P. (2008). Langmuir.

[cit13] Schwarz S., Lunkwitz K., Kessler B., Spiegler U., Killmann E., Jaeger W. (2000). Colloids Surf., A.

[cit14] Wang X., Guerin G., Wang H., Wang Y., Manners I., Winnik M. A. (2007). Science.

[cit15] Won Y.-Y., Davis H. T., Bates F. S. (1999). Science.

[cit16] Qiu H., Du V. A., Winnik M. A., Manners I. (2013). J. Am. Chem. Soc..

[cit17] Massey J., Power K. N., Manners I., Winnik M. A. (1998). J. Am. Chem. Soc..

[cit18] Geng Y., Discher D. E. (2005). J. Am. Chem. Soc..

[cit19] Won Y.-Y., Paso K., Davis H. T., Bates F. S. (2001). J. Phys. Chem. B.

[cit20] Gilroy J. B., Gaedt T., Whittell G. R., Chabanne L., Mitchels J. M., Richardson R. M., Winnik M. A., Manners I. (2010). Nat. Chem..

[cit21] Rupar P. A., Chabanne L., Winnik M. A., Manners I. (2012). Science.

[cit22] Petzetakis N., Dove A. P., O'Reilly R. K. (2011). Chem. Sci..

[cit23] Geng Y., Dalhaimer P., Cai S., Tsai R., Tewari M., Minko T., Discher D. E. (2007). Nat. Nanotechnol..

[cit24] Cai S., Vijayan K., Cheng D., Lima E. M., Discher D. E. (2007). Pharm. Res..

[cit25] Groschel A. H., Walther A., Lobling T. I., Schacher F. H., Schmalz H., Muller A. H. E. (2013). Nature.

[cit26] Kang Y., Pitto-Barry A., Maitland A., O'Reilly R. K. (2015). Polym. Chem..

[cit27] Moughton A. O., O'Reilly R. K. (2010). Chem. Commun..

[cit28] Garrett E. T., Pei Y., Lowe A. B. (2016). Polym. Chem..

[cit29] Pei Y., Dharsana N. C., Lowe A. B. (2015). Aust. J. Chem..

[cit30] Zhang W., D'Agosto F., Boyron O., Rieger J., Charleux B. (2011). Macromolecules.

[cit31] Rieger J., Grazon C., Charleux B., Alaimo D., Jerome C. (2009). J. Polym. Sci., Part A: Polym. Chem..

[cit32] Dan M., Huo F., Xiao X., Su Y., Zhang W. (2014). Macromolecules.

[cit33] Zhang W.-J., Hong C.-Y., Pan C.-Y. (2014). Macromolecules.

[cit34] Zhao W., Gody G., Dong S., Zetterlund P. B., Perrier S. (2014). Polym. Chem..

[cit35] Derry M. J., Fielding L. A., Armes S. P. (2015). Polym. Chem..

[cit36] Fielding L. A., Derry M. J., Ladmiral V., Rosselgong J., Rodrigues A. M., Ratcliffe L. P. D., Sugihara S., Armes S. P. (2013). Chem. Sci..

[cit37] Cai W., Wan W., Hong C., Huang C., Pan C. (2010). Soft Matter.

[cit38] He W. D., Sun X. L., Wan W. M., Pan C. Y. (2011). Macromolecules.

[cit39] Boisse S., Rieger J., Belal K., Di-Cicco A., Beaunier P., Li M.-H., Charleux B. (2010). Chem. Commun..

[cit40] Zhang X., Boisse S., Zhang W., Beaunier P., D'Agosto F., Rieger J., Charleux B. (2011). Macromolecules.

[cit41] Boisse S., Rieger J., Pembouong G., Beaunier P., Charleux B. (2011). J. Polym. Sci., Part A: Polym. Chem..

[cit42] Zhang W., D'Agosto F., Boyron O., Rieger J., Charleux B. (2012). Macromolecules.

[cit43] Zhang W., D'Agosto F., Dugas P.-Y., Rieger J., Charleux B. (2013). Polymer.

[cit44] Semsarilar M., Penfold N. J. W., Jones E. R., Armes S. P. (2015). Polym. Chem..

[cit45] Williams M., Penfold N. J. W., Lovett J. R., Warren N. J., Douglas C. W. I., Doroshenko N., Verstraete P., Smets J., Armes S. P. (2016). Polym. Chem..

[cit46] Ferguson C. J., Hughes R. J., Pham B. T. T., Hawkett B. S., Gilbert R. G., Serelis A. K., Such C. H. (2002). Macromolecules.

[cit47] Cunningham V. J., Alswieleh A. M., Thompson K. L., Williams M., Leggett G. J., Armes S. P., Musa O. M. (2014). Macromolecules.

[cit48] Warren N. J., Mykhaylyk O. O., Mahmood D., Ryan A. J., Armes S. P. (2014). J. Am. Chem. Soc..

[cit49] Zhang Q., Zhu S. (2015). ACS Macro Lett..

[cit50] Zhang B., Yan X., Alcouffe P., Charlot A., Fleury E., Bernard J. (2015). ACS Macro Lett..

[cit51] Derry M. J., Fielding L. A., Armes S. P. (2016). Prog. Polym. Sci..

[cit52] Lesage de la Haye J., Zhang X., Chaduc I., Brunel F., Lansalot M., D'Agosto F. (2016). Angew. Chem., Int. Ed..

[cit53] Boursier T., Georges S., Mosquet M., Rinaldi D., D'Agosto F. (2016). Polym. Chem..

[cit54] Warren N. J., Armes S. P. (2014). J. Am. Chem. Soc..

[cit55] Mable C. J., Warren N. J., Thompson K. L., Mykhaylyk O. O., Armes S. P. (2015). Chem. Sci..

[cit56] Yang P., Ratcliffe L. P. D., Armes S. P. (2013). Macromolecules.

[cit57] Blanazs A., Verber R., Mykhaylyk O. O., Ryan A. J., Heath J. Z., Douglas C. W., Armes S. P. (2012). J. Am. Chem. Soc..

[cit58] Verber R., Blanazs A., Armes S. P. (2012). Soft Matter.

[cit59] Simon K. A., Warren N. J., Mosadegh B., Mohammady M. R., Whitesides G. M., Armes S. P. (2015). Biomacromolecules.

[cit60] Thompson K. L., Mable C. J., Cockram A., Warren N. J., Cunningham V. J., Jones E. R., Verber R., Armes S. P. (2014). Soft Matter.

[cit61] Mitchell D. E., Lovett J. R., Armes S. P., Gibson M. I. (2016). Angew. Chem., Int. Ed..

[cit62] Penfold N. J. W., Lovett J. R., Warren N. J., Verstraete P., Smets J., Armes S. P. (2016). Polym. Chem..

[cit63] Lovett J. R., Warren N. J., Ratcliffe L. P. D., Kocik M. K., Armes S. P. (2015). Angew. Chem., Int. Ed..

[cit64] Fielding L. A., Lane J. A., Derry M. J., Mykhaylyk O. O., Armes S. P. (2014). J. Am. Chem. Soc..

[cit65] Pei Y., Sugita O. R., Thurairajah L., Lowe A. B. (2015). RSC Adv..

[cit66] Pei Y., Thurairajah L., Sugita O. R., Lowe A. B. (2015). Macromolecules.

[cit67] Penfold N. Lovett J. R. Verstraete P. Smets J. Armes S. P., Polym. Chem., 2016, 7 , 79 10.1039/C5PY01510C , , in the press .

[cit68] Bütün V., Lowe A. B., Billingham N. C., Armes S. P. (1999). J. Am. Chem. Soc..

[cit69] O'Reilly R. K., Hawker C. J., Wooley K. L. (2006). Chem. Soc. Rev..

[cit70] Guo A., Liu G., Tao J. (1996). Macromolecules.

[cit71] Chambon P., Blanazs A., Battaglia G., Armes S. P. (2012). Langmuir.

[cit72] Joralemon M. J., O'Reilly R. K., Hawker C. J., Wooley K. L. (2005). J. Am. Chem. Soc..

[cit73] Zhang Q., Remsen E. E., Wooley K. L. (2000). J. Am. Chem. Soc..

[cit74] Liu S., Weaver J. V. M., Tang Y., Billingham N. C., Armes S. P., Tribe K. (2002). Macromolecules.

[cit75] Sugihara S., Armes S. P., Blanazs A., Lewis A. L. (2011). Soft Matter.

[cit76] Lovett J. R., Ratcliffe L. P. D., Warren N. J., Armes S. P., Saunders B. R. (2016). Macromolecules.

[cit77] Smith A. E., Xu X., Abell T. U., Kirkland S. E., Hensarling R. M., McCormick C. L. (2009). Macromolecules.

[cit78] Liu G., Qiu Q., Shen W., An Z. (2011). Macromolecules.

[cit79] Semsarilar M., Ladmiral V., Blanazs A., Armes S. P. (2012). Langmuir.

[cit80] Semsarilar M., Ladmiral V., Blanazs A., Armes S. P. (2013). Langmuir.

[cit81] Williams M., Penfold N. J. W., Armes S. P. (2016). Polym. Chem..

[cit82] Lopez-Oliva A. P., Warren N. J., Rajkumar A., Mykhaylyk O. O., Derry M. J., Doncom K. E. B., Rymaruk M. J., Armes S. P. (2015). Macromolecules.

[cit83] Buckingham S. A., Garvey C. J., Warr G. G. (1993). J. Phys. Chem..

[cit84] Ekwall P., Mandell L., Solyom P. (1971). J. Colloid Interface Sci..

[cit85] McFarlane N. L., Wagner N. J., Kaler E. W., Lynch M. L. (2010). Langmuir.

[cit86] Lafuma F., Wong K., Cabane B. (1991). J. Colloid Interface Sci..

[cit87] Mabire F., Audebert R., Quivoron C. (1984). J. Colloid Interface Sci..

[cit88] Blanazs A., Madsen J., Battaglia G., Ryan A. J., Armes S. P. (2011). J. Am. Chem. Soc..

