# Supplementary material for: Cross-linked cationic diblock copolymer worms are superflocculants for micrometer-sized silica particles
Source: Chem Sci. 2016 Sep 13;7(12):6894–904. doi: 10.1039/c6sc03732a (PMC5450592; doi:10.1039/c6sc03732a)
Supplement: Supplementary file 1 [file SC-007-C6SC03732A-s001.pdf]

Electronic Supplementary Material (ESI) for Chemical Science.
This journal is © The Royal Society of Chemistry 2016

Supporting Information for:

Cross-Linked Cationic Diblock Copolymer Worms are Superflocculants for Micrometer-sized Silica Particles

Nicholas J. W. Penfold, Yin Ning, Steven P. Armes,
Pierre Verstraete and Johan Smets

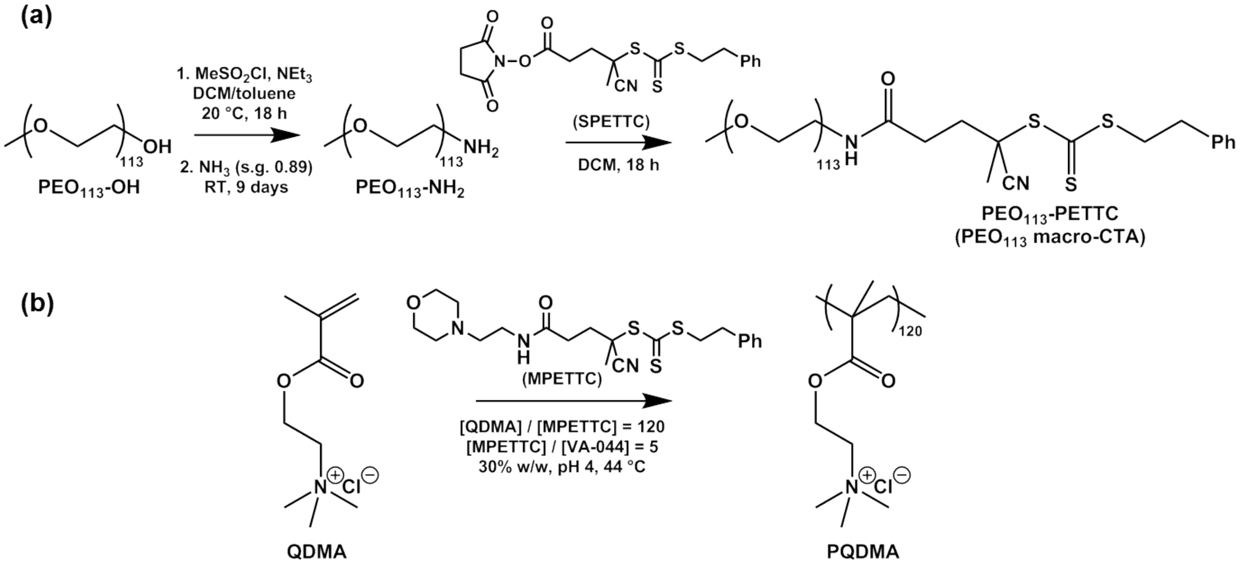

Scheme S1. Synthesis details for (a) preparation of monoaminated PEO_{113}-NH_2 from its PEO_{113} monomethyl ether precursor followed by amidation reaction between PEO_{113}-NH_2 and SPETTC and (a) RAFT aqueous solution polymerization of QDMA at pH 4 using MPETTC as the RAFT agent. MPETTC and SPETTC were synthesized using protocols reported elsewhere.[62]

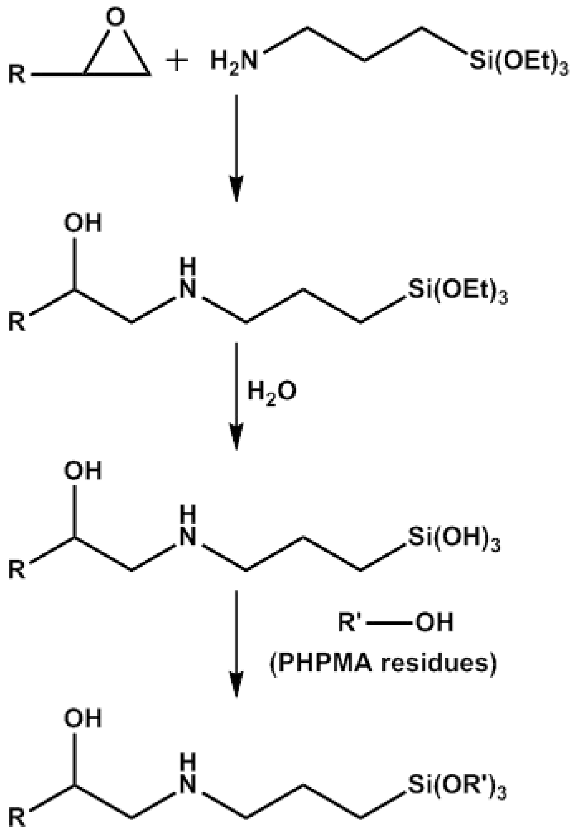

Scheme S2. Suggested cross-linking chemistry for the formation of the cross-linked cationic diblock copolymer worms described in this work. Epoxy groups on glycidyl methacrylate residues are ring-opened via reaction with 3-aminopropyl triethoxysilane with simultaneous hydrolysis to form pendent silanol groups that condense both with each other and with secondary hydroxyl groups on HPMA residues.[76]

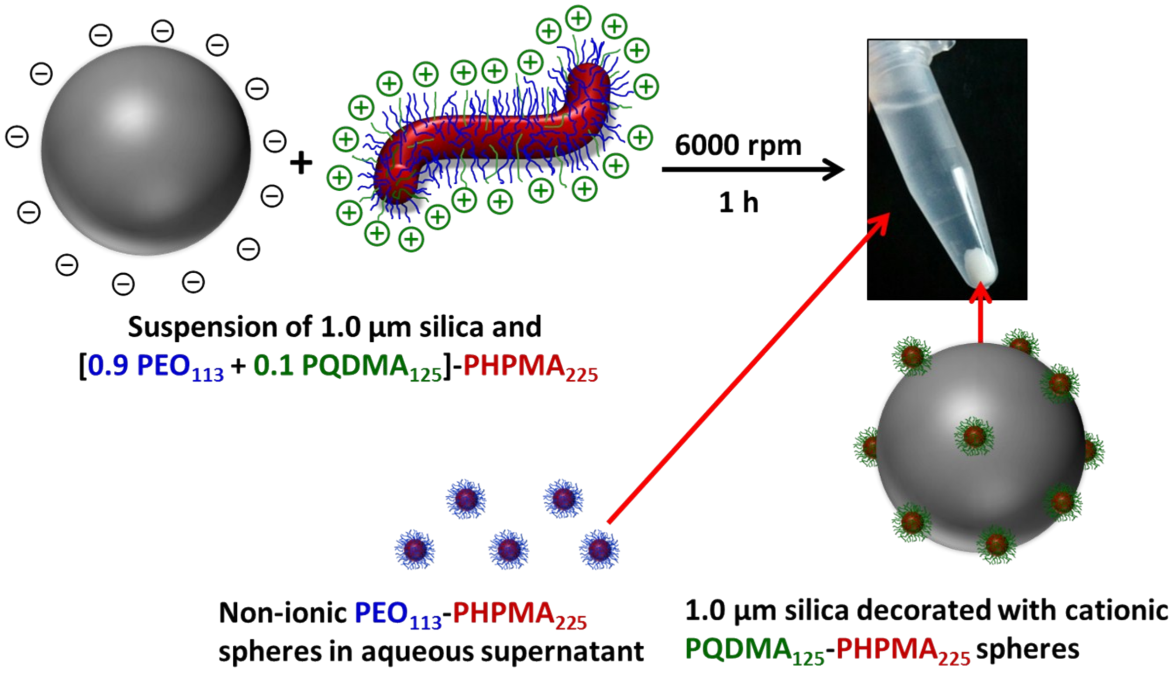

Scheme S3. Schematic representation of the suggested *in situ* break-up of linear (0.9 PEO_{113} + 0.1 $PQDMA_{125}$)-$PHPMA_{225}$ diblock copolymer worms in the presence of 1.0 µm silica particles to form two distinct populations of (mainly) non-ionic PEO_{113}-$PHPMA_{225}$ and (mainly) cationic $PQDMA_{125}$-$PHPMA_{225}$ diblock copolymer nanoparticles. Images shown are not to scale.

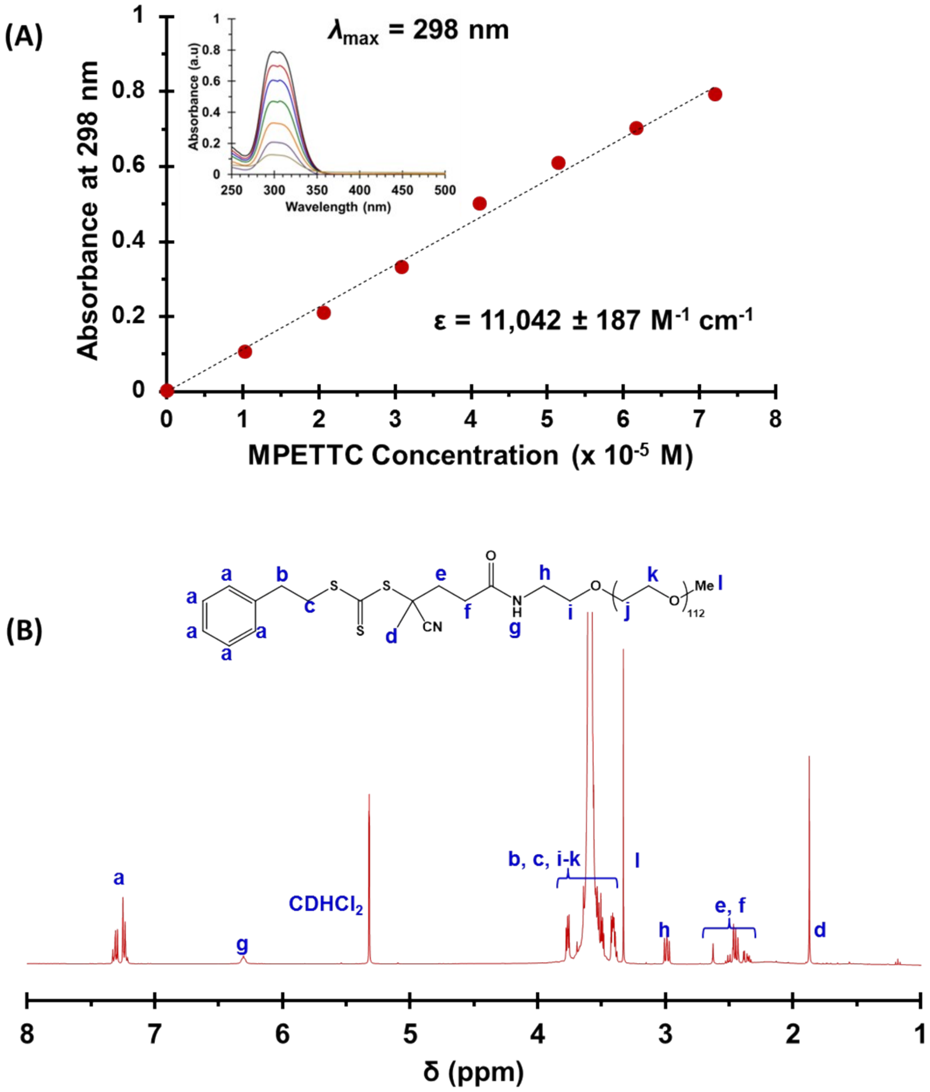

Figure S1. (A) Beer-Lambert plot for MPETTC RAFT agent in methanol to calculate PEO_{113}-PETTC functionality by UV spectroscopy and (B) assigned ^1H NMR spectra obtained for PEO_{113}-PETTC macro-CTA in CD_2Cl_2 to calculate the mean degree of amidation.

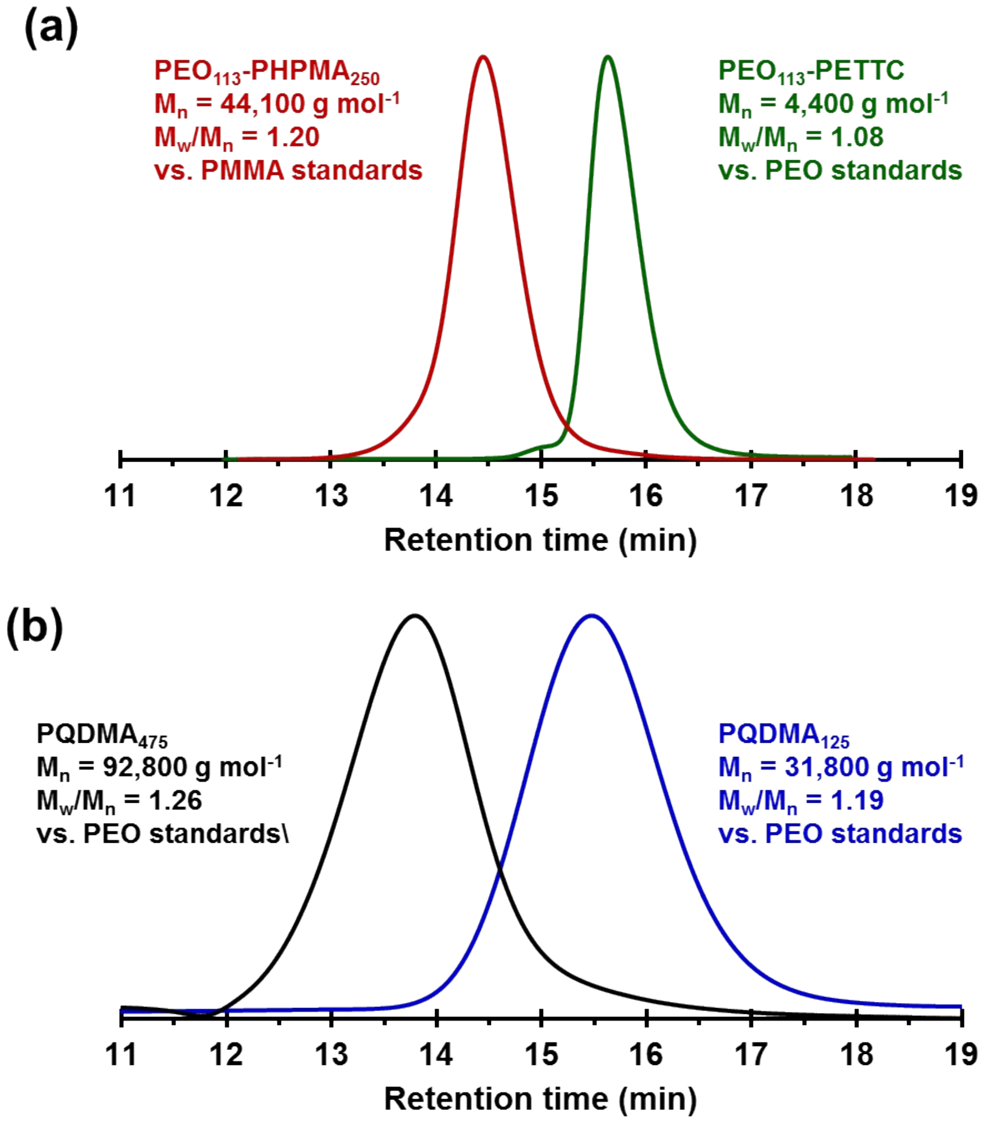

Figure S2. (a) THF GPC chromatograms for both PEO_{113} macro-CTA (vs. PEO standards) and PEO_{113}-$PHPMA_{250}$ diblock copolymer (vs. PMMA standards). (b) Aqueous GPC chromatograms (vs. PEO standards) for both $PQDMA_{125}$ macro-CTA and $PQDMA_{475}$, after a self-blocking experiment using 350 units of QDMA.

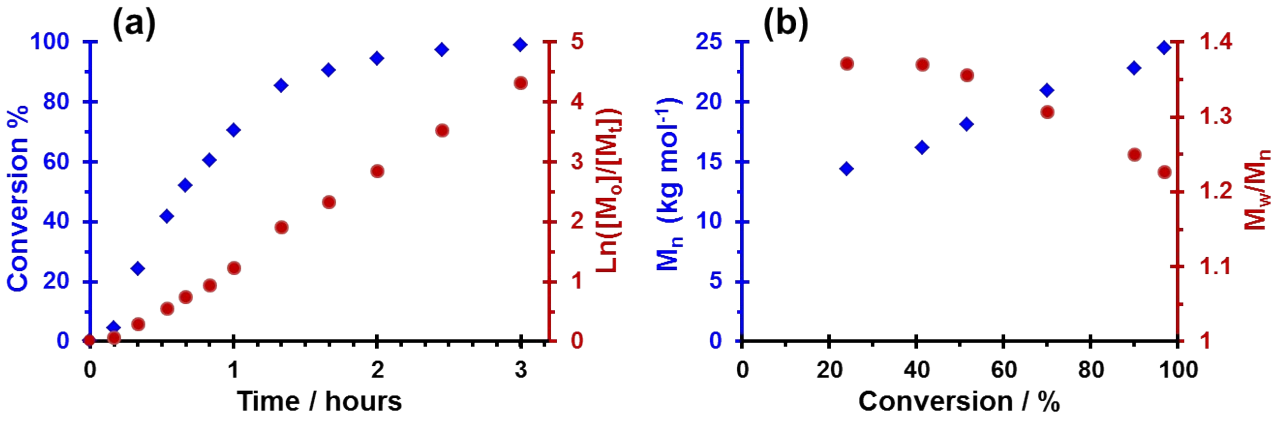

Figure S3. (a) Conversion and semi-logarithmic curves vs. time and (b) number-average molecular weight (M_n) and dispersity (M_w/M_n) vs. conversion plots for the RAFT aqueous solution polymerization of [2-(methacryloyloxy)ethyl] trimethylammonium chloride at 30% w/w solids, pH 4 and 44 °C using a [MPETTC]/[VA-044] molar ratio of 5.0, while targeting a DP of 120. Monomer conversions were calculated from ^{1}H NMR spectra recorded in CD_3OD by comparing the integrated aromatic MPETTC signals at 7.2-7.4 ppm to that of the QDMA monomer vinyl signal at 6.2 ppm. Molecular weight data were assessed by aqueous GPC analysis at pH 2 using a series of near-monodisperse poly(ethylene oxide) (PEO) standards.

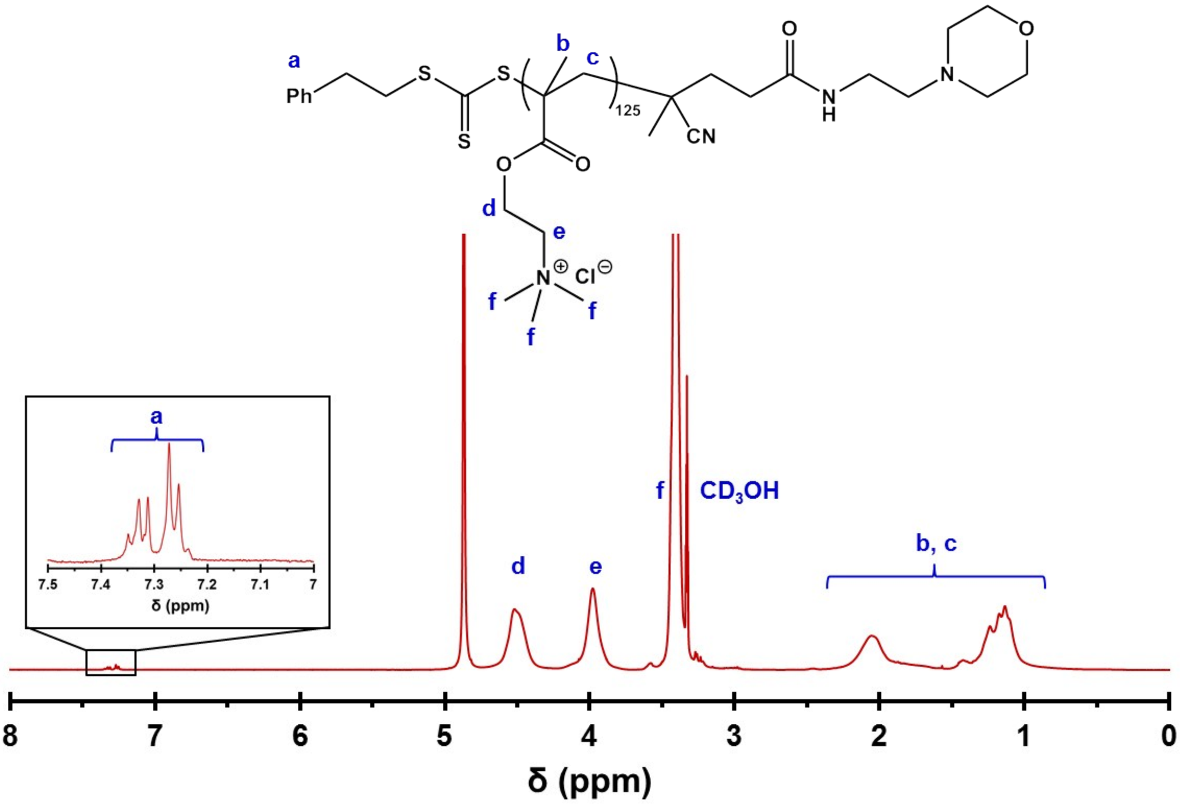

Figure S4. Assigned ^{1}H NMR spectrum in CD_3OD for poly[2-(methacryloyloxy)ethyl] trimethylammonium chloride synthesized by RAFT aqueous solution polymerization using MPETTC RAFT agent at pH 4. The degree of polymerization was calculated by comparing the integrated aromatic signals assigned to the RAFT agent at 7.2-7.4 ppm to those of the methacrylic polymer backbone at 0.8-2.4 ppm.

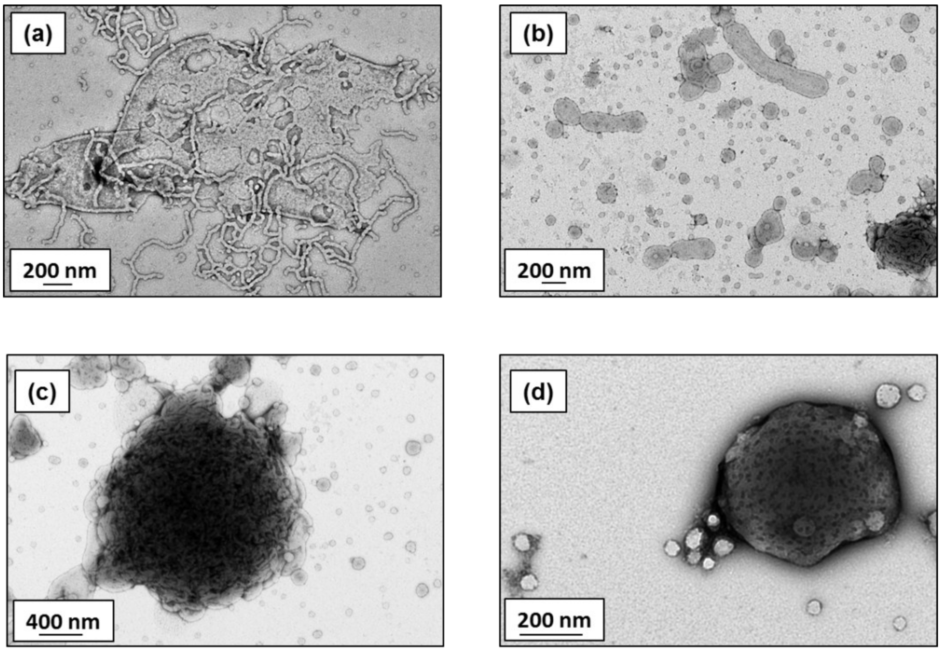

Figure S5. Representative TEM images obtained for a series of (0.95 PEO_{113} + 0.05 $PQDMA_{125}$)-$PHPMA_z$ diblock copolymer nanoparticles where (a) z = 250, (b) z = 400, (c) z = 500 and (d) z = 600.

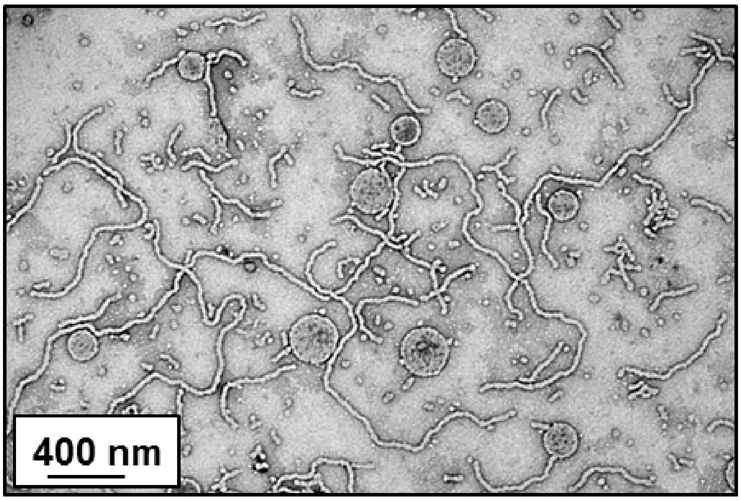

Figure S6. Representative TEM images of (0.9 PEO_{113} + 0.1 $PQDMA_{125}$)-(P(HP MA_{168}-*stat*-$GlyMA_{42}$)) worm/vesicle mixed phase (total core DP = 225).

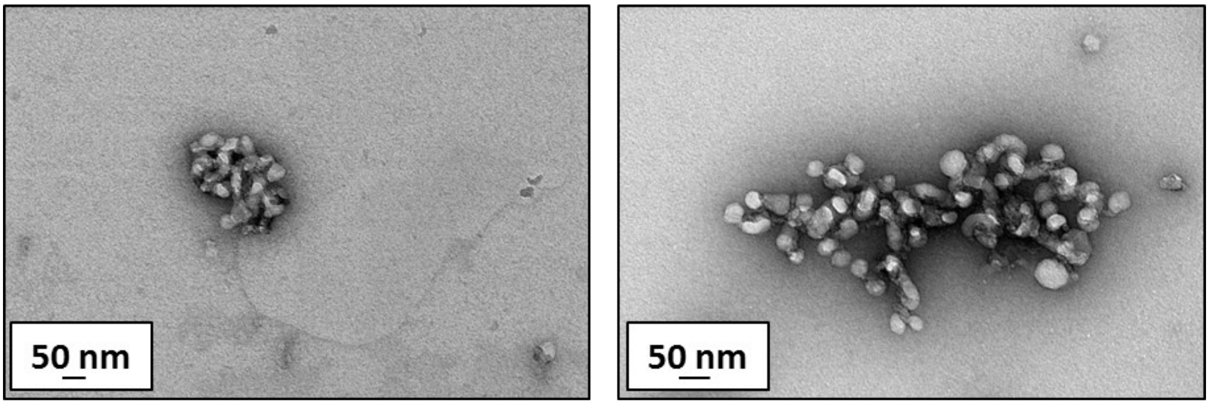

Figure S7. Representative TEM images of copolymer nanoparticles present in the dried aqueous supernatant obtained after the centrifugation of a mixture of 1.0 µm silica particles and linear (0.9 PEO_{113} + 0.1 $PQDMA_{125}$)-$PHPMA_{225}$ cationic block copolymer worms prepared at a surface density of 4.7 mg m^{-2}.

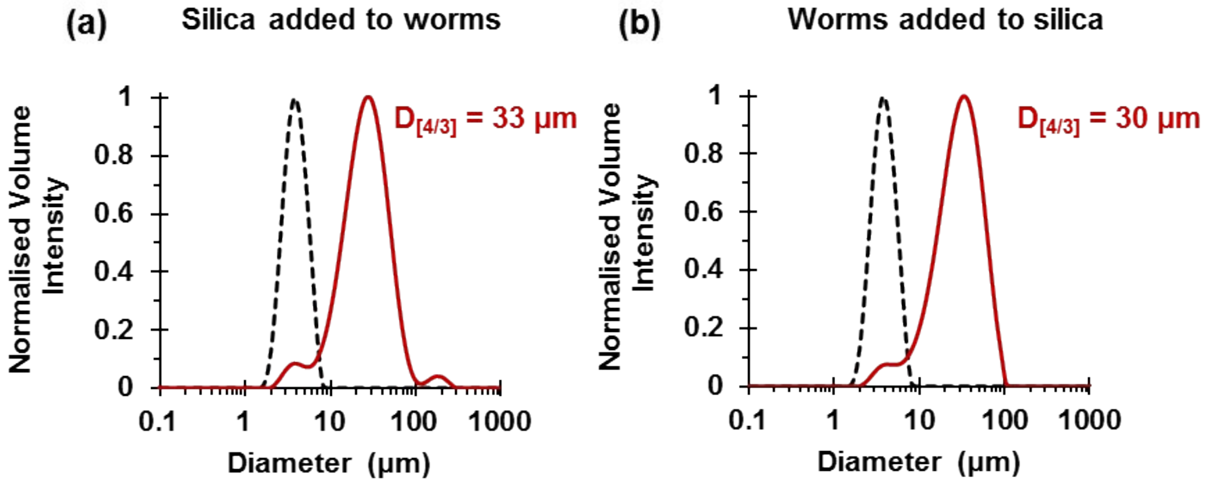

Figure S8. Volume-average particle size distributions obtained via laser diffraction for cross-linked cationic worms adsorbed onto 4 µm silica particles at a nominal adsorbed amount of 88 mg m^{-2} where (a) the silica is added to the worms and (b) the worms are added to the silica particles. The black dotted traces represent the volume-average particle size distribution obtained for the pristine 4 µm silica particles in the absence of any worms.

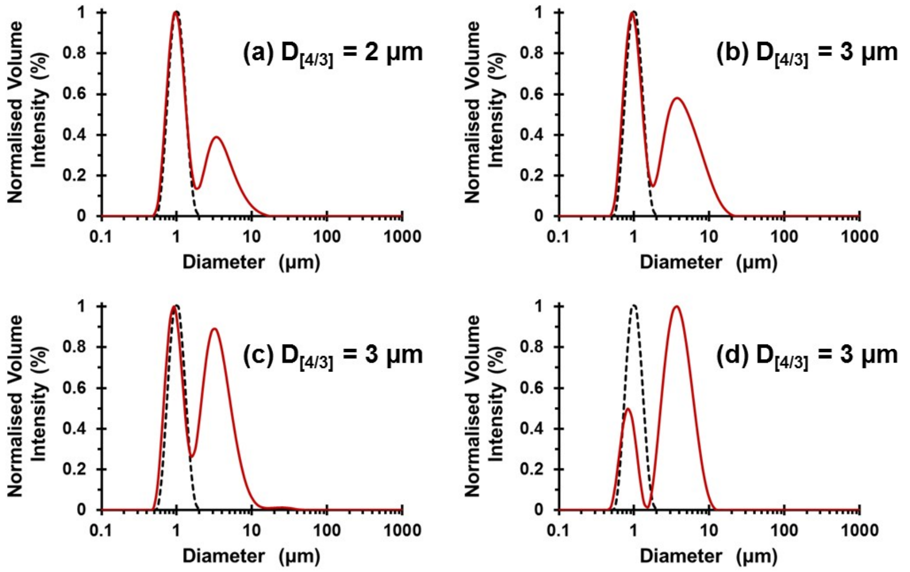

Figure S9. Volume-average particle size distributions obtained via laser diffraction for the attempted flocculation of 1.0 µm silica at pH 9 using poly(ethylene glycol) (M_w = 4,000,000 g mol^{-1}) at surface densities of (a) 2.0 mg m^{-2}, (b) 4.7 mg m^{-2}, (c) 17.2 mg m^{-2} and (d) 34.3 mg m^{-2}.

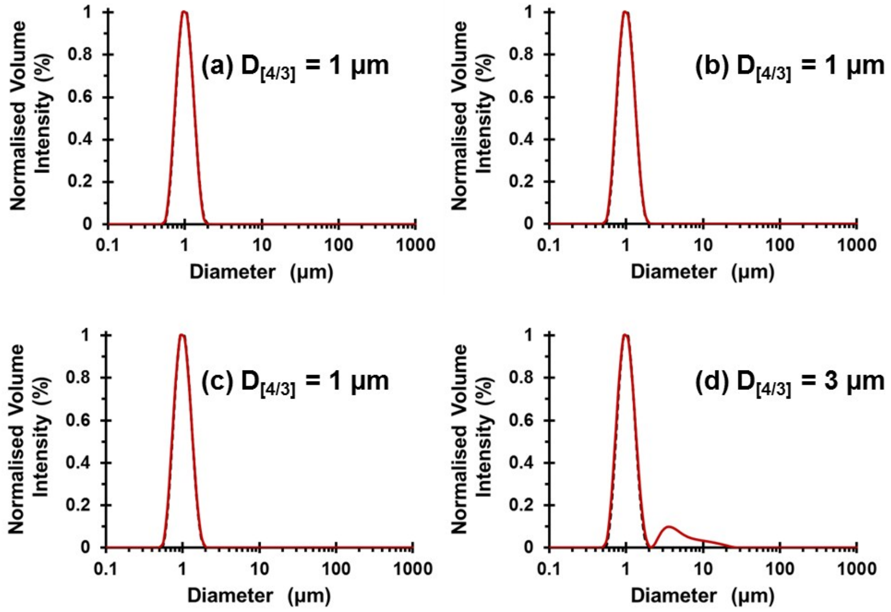

Figure S10. Volume-average particle size distributions obtained via laser diffraction for the attempted flocculation of 1.0 µm silica at pH 9 using poly(acrylamide) (M_w = 6,000,000 g mol^{-1}) at surface densities of (a) 2.0 mg m^{-2}, (b) 4.7 mg m^{-2}, (c) 17.2 mg m^{-2} and (d) 34.3 mg m^{-2}.

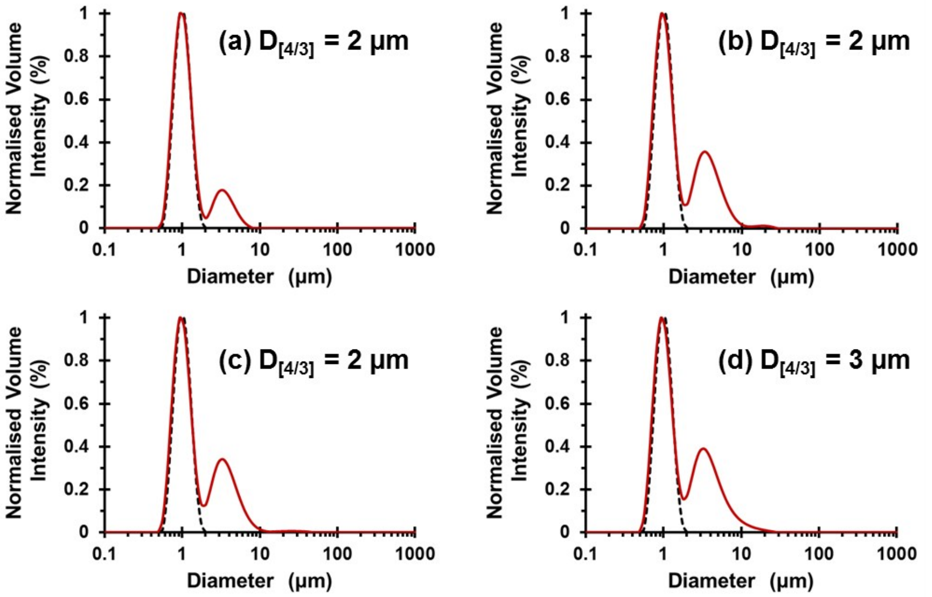

Figure S11. Volume-average particle size distributions obtained via laser diffraction for the attempted flocculation of 1.0 µm silica at pH 9 using poly(vinylpyrrolidone) (M_w = 1,300,000 g mol^{-1}) at surface densities of (a) 2.0 mg m^{-2}, (b) 4.7 mg m^{-2}, (c) 17.2 mg m^{-2} and (d) 34.3 mg m^{-2}.

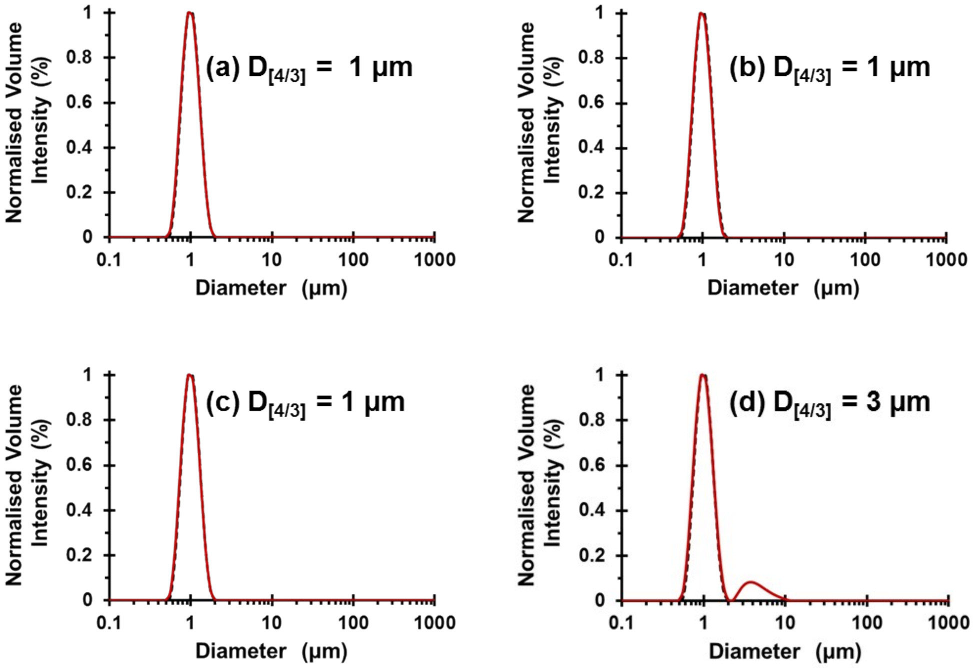

Figure S12. Volume-average particle size distributions obtained via laser diffraction for the attempted flocculation of 1.0 µm silica at pH 9 using poly(diallyldimethylammonium chloride) (M_w = 500,000 g mol^{-1}) at surface densities of (a) 2.0 mg m^{-2}, (b) 4.7 mg m^{-2}, (c) 17.2 mg m^{-2} and (d) 34.3 mg m^{-2}.

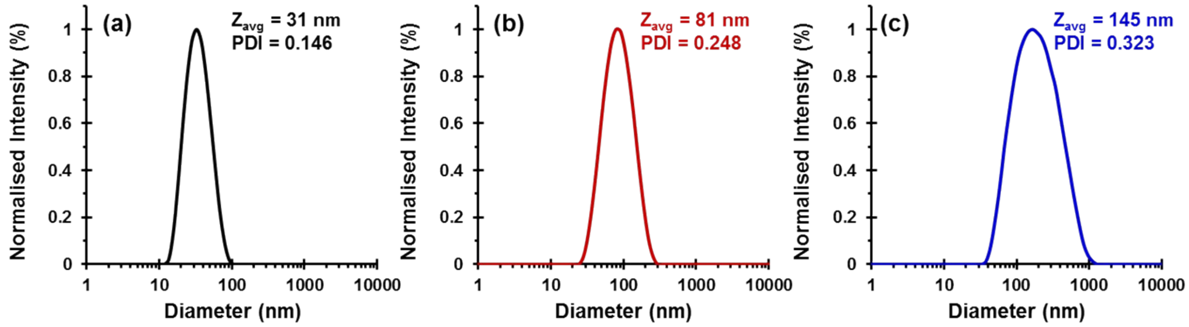

Figure S13. Intensity-average particle size distributions obtained via dynamic light scattering for (a) Bindzil 2040 silica sols alone and the silica sols flocculated by (b) poly(ethylene oxide) and (c) poly(diallyldimethylammonium chloride) at a surface density of 5.2 mg m^{-2}.

Table S1. Summary of intensity-average diameter, zeta potential and derived count rate data obtained for 0.1% w/w dispersions of linear (0.9 PEO_{113} + 0.1 $PQDMA_{125}$)-$PHPMA_{225}$ and core cross-linked (0.9 PEO_{113} + 0.1 $PQDMA_{125}$)-P($HPMA_{160}$-*stat*-$GlyMA_{40}$) block copolymer worms either at pH 9, after serial dilution of the as-prepared worms using methanol or in the presence of 0.1% w/w CTAB. All dispersions were analyzed in the presence of 1 mM KCl.

Worm type	Intensity-average diameter / nm (PDI)	Zeta potential / mV (1 SD)	DLS derived-count rate / kcps	Normalized Intensity
Cross-linked worms at pH 9	216 (0.244)	+34 (± 2)	6.0×10^4	100
Cross-linked worms in methanol	240 (0.239)	-	5.4×10^4	90
Cross-linked worms at pH 9 plus 0.1% CTAB	212 (0.292)	+33 (± 4)	5.9×10^4	97
Linear worms at pH 9	212 (0.292)	+35 (± 2)	5.9×10^4	100
Linear worms in methanol	78 (0.208)	-	1.4×10^2	0.2
Linear worms at pH 9 plus 0.1% CTAB	48 (0.762)	+38 (± 8)	9.3×10^2	1.6

Table S2. Intensity-average diameters (nm) obtained by dynamic light scattering for the attempted flocculation of 31 nm silica sols at pH 9 using four commercially available polymers. $[silica]_0$ = 0.05% w/w.

Commercial Polymer	M_w (g mol^{-1})	Intensity-average diameter (nm)					
		0.6 mg m^{-2}	1.4 mg m^{-2}	2.6 mg m^{-2}	5.2 mg m^{-2}	7.7 mg m^{-2}	10.3 mg m^{-2}
PEO	4,000,000	57	60	76	81	78	84
PA	6,000,000	34	55	41	39	70	76
PVP	1,300,000	52	57	60	59	66	66
PDADMAC	500,000	181	149	142	145	135	165

Experimental Details

Materials

[2-(Methacryloyloxy)ethyl] trimethylammonium chloride (QDMA, 80% w/w aqueous solution), poly(ethylene oxide) monomethyl ether (PEO, mean DP = 113), glycidyl methacrylate (GlyMA, 97%), 3-aminopropyl triethoxysilane (APTES, >98%), triethylamine (>99%), methanesulfonyl chloride (>99%) were all purchased from Sigma Aldrich (UK) and used as received. Deuterated methanol (CD_3OD; D, 99.8%) and dichloromethane (CD_2Cl_2; D, 99.9%) were purchased from Cambridge Isotopes Laboratories Ltd. 2-Hydroxypropyl methacrylate (HPMA; 97%) was purchased from Alfa Aesar (UK) and used as received. VA-044 was purchased from Wako Chemicals Ltd. All solvents were purchased from either VWR International or Sigma Aldrich and were HPLC-grade quality. Commercial polymers: Poly(ethylene glycol) (M_w = 4,000,000 g mol^{-1}) and poly(acrylamide) (M_w = 6,000,000 g mol^{-1}) were purchased from Polysciences Inc, Warrington, PA, USA. Poly(vinylpyrrolidone) (M_w = 1,300,000 g mol^{-1}) and poly(diallyldimethylammonium chloride) (M_w = 500,000 g mol^{-1}) were purchased from Sigma Aldrich. Commercial silica particles: AngstromSphere silica particles of nominal 1.0 µm & 4 µm diameters were purchased from Fiber Optic Center Inc. (MA) and used as received. Bindzil 2040 silica particles of diameter 31 nm (measured by DLS) were kindly donated from AkzoNobel.

Methods

Synthesis of mono-aminated poly(ethylene glycol)$_{113}$-NH_2

Poly(ethylene glycol) monomethyl ether (50.0 g, 10.0 mmol, M_n = 5000 g mol^{-1}) was dissolved in toluene (500 ml) and this solution was azeotropically distilled to remove approximately 300 ml of solvent. After cooling to room temperature, anhydrous dichloromethane (200 ml) was added, then triethylamine (6.00 g, 59.3 mmol) was added dropwise. Subsequently, methanesulfonyl chloride (6.79 g, 59.3 mmol) was added dropwise and the reaction solution was stirred under N_2 for 18 hours. The white ammonium chloride salt was removed by filtration and the organic layer was concentrated under vacuum, then precipitated into diethyl ether. The off-white precipitate was collected by filtration and dried in a 30 °C vacuum oven to yield PEO$_{113}$-OMs (48.8 g, 9.76 mmol). PEO$_{113}$-OMs was

dissolved in 2 L of 35% aqueous ammonia over a 7 h period. The lid was sealed and the solution was stirred at 20 °C for 6 days. The lid was removed and stirred for a further 3 days to remove excess ammonia. The pH was increased to pH 13 using NaOH and the polymer was extracted into dichloromethane (3 x 150 ml). The organic layers were washed with brine and dried over magnesium sulfate. After concentration under vacuum, the polymer was precipitated into diethyl ether and PEO_{113}-NH_2 (45.1 g, 9.02 mmol) was isolated by filtration and dried under vacuum, with a yield of 90%. The primary amine end-group was confirmed by ^{1}H NMR spectroscopy as the triplet signal of the methylene protons adjacent to the amine was observed at δ 2.9 ppm. Comparison of this integrated triplet signal, relative to that of the methyl ether end group, indicated an end-group functionality of 96 %.

Synthesis of poly(ethylene glycol)$_{113}$-PETTC macro-CTA

All glassware was dried in a 120 °C oven overnight then flame-dried under vacuum before use to remove trace water. SPETTC was synthesized according to previous protocol.[43] SPETTC (4.45 g, 10.2 mmol) was dissolved in anhydrous dichloromethane (20 ml) in a 250 ml two-necked round-bottomed flask equipped with a pressure-equalizing dropping funnel. PEO_{113}-NH_2 (45.1 g, 9.02 mmol) was dissolved in anhydrous dichloromethane (100 ml) and added to the dropping funnel via cannula transfer under N_2. The PEO_{113}-NH_2 solution was added dropwise to the yellow SPETTC solution over approximately 1 hour and stirred overnight at room temperature. The yellow polymer solution was precipitated into diethyl ether (1500 ml) and the yellowish polymer was isolated by vacuum filtration and dried in a 30 °C vacuum oven to yield PEO_{113}-PETTC (42.1 g, 7.88 mmol) in 87% yield. ^{1}H NMR spectroscopy confirmed conjugation of the RAFT agent to the mono-aminated PEO_{113} and comparison of the aromatic integrals at δ 7.1 – 7.4 ppm with the triplet at δ 2.9 ppm indicated a degree of PETTC functionality of 96%. UV spectroscopy indicated an end-group functionality of 94%. THF GPC indicated an M_n = 4,400 g mol^{-1}, M_w/M_n = 1.08 vs. PEO standards

Synthesis of poly([2-(methacryloyloxy)ethyl]trimethylammonium chloride macro-CTA by RAFT aqueous solution polymerization

MPETTC RAFT agent was synthesized according to previous reports.[30] A 100 ml round-bottom flask was charged with MPETTC (0.237 g, 0.524 mmol) and ([2-(methacryloyloxy)ethyl] trimethylammonium chloride monomer (QDMA) (16.4 g of 80% aqueous solution, 13.1 g QDMA monomer, 63.0 mmol, target DP = 120) and stirred for 5 minutes. Then 2,2'-azobis[2-(2-imidazolin-2-yl)propane]dihydrochloride (VA-044, 34.0 mg, 0.105 mmol) and H_2O (27.9 g) were added to afford a 30% w/w cloudy yellow solution. The pH was lowered to 4.0 by the careful addition of 1 M HCl and the cloudy solution became transparent and was stirred for 5 min. The yellow solution was degassed under N_2 for 30 min in an ice/water slurry. The sealed reaction vessel was immersed in a preheated 44 °C oil bath for 3 h. Polymerization was quenched by exposure to air and cooling to room temperature. Monomer conversion was calculated to be 96% conversion by ^{1}H NMR. Purification and isolation of the polymer was achieved by precipitation into excess acetonitrile (3 x 500 ml), dissolution into deionized water, removal of residual acetonitrile under vacuum and then freeze-drying overnight. ^{1}H NMR spectroscopy confirmed a DP of 125 by comparison of the integral of the aromatic end-group signal at δ 7.1 – 7.4 ppm to that of the methacrylic backbone at δ 0.4 – 2.5 ppm, indicating a RAFT agent efficiency of

92% . Aqueous GPC analysis of the $PQDMA_{125}$ macro-CTA indicated an M_n = 31,800 g mol^{-1} and M_w/M_n = 1.19 against PEO standards.

Synthesis of linear (poly(ethylene glycol) + poly([2-(methacryloyloxy)ethyl]trimethylammonium chloride))-poly(2-hydroxypropyl methacrylate) diblock copolymer nano-objects by RAFT aqueous dispersion of 2-hydroxypropyl methacrylate

In a typical protocol for the synthesis of linear cationic nano-objects (0.9 PEO_{113} + 0.1 $PQDMA_{125}$)-$PHPMA_{225}$ at 20% solids: A 7 mL sample vial was charged with $PQDMA_{125}$ macro-CTA (77.7 mg, 2.94 µmol), PEO_{113}-PETTC macro-CTA (0.152 g, 26.4 µmol), VA-044 (3.17 mg, 9.80 µmol), HPMA (0.954 g, 6.62 mmol) and water (4.75 g) to afford a 20% w/w solution. The sealed vial was degassed under N_2 in an ice/water slurry for 20 minutes placed in a preheated 50 °C oil bath for 4 h. Polymerization was quenched by exposure to air and cooling 20 °C. ^{1}H NMR spectroscopy was used to determine final monomer conversion and TEM was used to determine the copolymer morphology. A series of block copolymer nano-particles were synthesized by variation of the molar ratio of PEO_{113}-PETTC to $PQDMA_{125}$ and the target degree of polymerization of the PHPMA block at a fixed concentration of 20% solids to generate a phase diagram.

Synthesis of core cross-linked (poly(ethylene glycol)+poly([2-(methacryloyloxy)ethyl]trimethylammonium chloride))-(poly(2-hydroxypropyl methacrylate)-*stat*-poly(glycidyl methacrylate)) copolymers worms by RAFT aqueous dispersion of 2-hydroxypropyl methacrylate and glycidyl methacrylate

In a typical protocol for the synthesis of cross-linked cationic worms (0.9 PEO_{113} + 0.1 $PQDMA_{125}$)-(P($HPMA_{160}$-*stat*-$GlyMA_{40}$)) at 20% solids: A 14ml sample vial was charged with $PQDMA_{125}$ macro-CTA (0.0801 g, 3.03 µmol), PEO_{113} macro-CTA (0.157 g, 27.3 µmol), VA-044 (3.30 mg, 10.2 µmol), HPMA (0.701 g, 4.86 mmol), GlyMA (0.173 g, 1.22 mmol) and deionized water (4.46 g) to afford a 20% w/w solution. The sealed vial was degassed under N_2 in an ice/water slurry for 20 minutes placed in a preheated 50 °C oil bath for 4 h. Polymerization was quenched by exposure to air and cooling 20 °C. TEM was used to determine a worm-like morphology. This worm-gel was diluted to 7.5% w/w by the addition of deionized water (9.28 g) and gently stirred for 24 h. (3-aminopropyl)triethoxysilane (0.269 g, 0.284 mL, 1.22 mmol, [PGlyMA]/[APTES] = 1) was added to the worm dispersion and stirred overnight. These core cross-linked cationic diblock copolymer worms were analyzed by DLS and TEM to assess stability in methanolic and cetyltrimethylammonium bromide solutions,

Surfactant Challenge

In a typical surfactant challenge, 1.0 mL of a pH 9, 0.20 % w/w aqueous dispersion of core cross-linked (0.9 PEO_{113} + 0.1 $PQDMA_{125}$)-(P($HPMA_{160}$-*stat*-$GlyMA_{40}$)) cationic worms was mixed with 1.0 mL of a 0.20 % w/w CTAB solution (prepared at pH 9 using an ultrasonic bath to aid surfactant dissolution) to give an aqueous solution containing 0.1% w/w copolymer and 0.1% w/w CTAB, followed by stirring for 24 h at 20 ºC. The resulting copolymer/surfactant dispersion was analyzed by DLS and TEM without further dilution.

Flocculation study of 1.0 µm, 4 µm and 31 nm silica particles using linear and cross-linked cationic block copolymer worms

In a typical flocculation experiment, 2.0 mL of a 2.0 % w/w dispersion of 1.0 µm silica spheres at pH 9 was added dropwise to 2.0 mL of a 0.028 % w/w dispersion of either linear or core cross-linked cationic diblock copolymer worms at pH 9 and gently stirred for 24 h at 20 ºC. The initial polymer concentration was varied (while keeping $[silica]_0$ at 2.0 % w/w) to change the surface densities of the worms on the 1.0 µm silica surface. In addition, the flocculated dispersion was centrifuged at 6000 rpm for 60 min and any nanoparticles remaining in the aqueous supernatant were analyzed by DLS and TEM. In separate experiments, 2.0 mL of 8% w/w of 4 µm silica dispersion was added to 2.0 mL of core cross-linked worm dispersions at pH 9. The resulting copolymer/silica dispersion was analyzed by laser diffraction and SEM to assess its degree of flocculation.

^{1}H NMR Spectroscopy

All NMR spectra were recorded at 298 K using a 400 MHz Bruker AV3-HD spectrometer in CD_3OD or CD_2Cl_2. Sixty-four scans were averaged per spectrum. All chemical shifts are reported in ppm (δ).

Dynamic Light Scattering

All DLS measurements were recorded at 20 °C using a Malvern Instruments Zetasizer Nano series instrument equipped with a 4 mW 633 nm He−Ne laser and an avalanche photodiode. Aqueous electrophoresis measurements were conducted in the presence of 1 mM KCl. The dispersion pH was adjusted as required with either 1 M or 0.1 M HCl or KOH.

Laser Diffraction

Laser diffraction studies were performed on a Malvern Mastersizer 3000 instrument was equipped with a Hydro EV dispersion unit and set at a stirring rate of 2000 rpm. A HeNe laser operating at 633 nm and a solid-state blue laser operating at 466 nm were used to analyse each silica/polymer dispersion to assess the degree of flocculation.

Aqueous Gel Permeation Chromatography

0.5 % w/w copolymer solutions were analysed in an acidic aqueous buffer (pH 2) containing 0.5 M acetic acid, 0.3 M NaH_2PO_4 and acidified with concentrated HCl at a flow rate of 1.0 ml min^{-1}. The GPC set-up comprised an Agilent 1260 Infinity series degasser and pump, an Agilent PL Aquagel-OH 30 8 µm column and a Agilent PL Aquagel-OH 20 5 µm column for PQDMA kinetic studies. This column set up was calibrated with PEO standards

ranging from 400 g mol^{-1} to 134,300 g mol^{-1}. For analysis of the $PQDMA_{125}$ macro-CTA and its self-blocking experiment, the column set-up comprised of two Agilent PL Aquagel-OH 30 8 µm columns in series. This column set-up was calibrated with PEO standards from 1,080 g mol^{-1} to 905,000 g mol^{-1}. In both cases, a refractive index detector was used at a flow rate of 1 mL min^{-1} and 30 °C.

THF Gel Permeation Chromatography

0.5% w/w copolymer solutions were prepared in THF using toluene (10 µL per mL) as the flow rate marker. GPC measurements were conducted using a THF eluent containing 2.0% v/v triethylamine and 0.05% w/v butylhydroxytoluene (BHT) at a 30 °C, flow rate of 1.0 mL min^{-1}. The GPC set-up comprised a two PLgel 5 µm Mixed C columns in series and a WellChrom K-2301 RI detector operating at 950 ± 30 nm. A series of ten near-monodisperse poly(methyl methacrylate) standards (M_p values from 1,280 g mol^{-1} to 330,000 g mol^{-1}) were used for column calibration.

Centrifugation

Copolymer/silica dispersions (1.5 mL) were centrifuged at 6000 rpm for 1 h using a Heraeus Biofuge Pico centrifuge. The aqueous supernatant was analyzed by DLS, aqueous electrophoresis and TEM. The sedimented silica particles were redispersed and analyzed by aqueous electrophoresis.

Helium Pycnometry

The solid-state density of the silica spheres was measured using a Micromeritics AccuPyc 1330 helium pycnometer at 20 °C.

Transmission Electron Microscopy

Copper/palladium grids were surface-coated in-house to produce a thin film of amorphous carbon and then plasma glow-discharged for 20 seconds to give a hydrophilic surface. Freshly-prepared 0.1% w/v aqueous dispersions (10 µL) were onto the hydrophilic grid for 15 seconds, blotted to remove excess sample and then negatively stained with uranyl formate solution (0.75% w/v) for a further 15 seconds. Excess stain was removed by blotting and carefully dried with a vacuum house. TEM grids were imaged using a FEI Tecnai Spirit 2 microscope fitted with an Orius SC1000B camera operating at 80 kV.

Scanning Electron Microscopy

Silica or copolymer/silica dispersions were placed on a glass slide and dried overnight. The silica or copolymer/silica glass slides were mounted onto SEM studs using adhesive conducting pads and then gold-coated prior to analysis. Imaging was performed using an Inspect F microscope operating at 15 kV.
